# Identification and characterization of new proteins crucial for bacterial spore resistance and germination

**DOI:** 10.3389/fmicb.2023.1161604

**Published:** 2023-04-11

**Authors:** Benjamin Yu, Julia Kanaan, Hannah Shames, James Wicander, Makunda Aryal, Yunfeng Li, George Korza, Stanley Brul, Gertjan Kramer, Yong-qing Li, Frank C. Nichols, Bing Hao, Peter Setlow

**Affiliations:** ^1^Department of Molecular Biology and Biophysics, UConn Health, Farmington, CT, United States; ^2^Department of Physics, East Carolina University, Greenville, NC, United States; ^3^Molecular Biology and Microbial Food Safety, University of Amsterdam, Amsterdam, Netherlands; ^4^Laboratory for Mass Spectrometry of Biomolecules, Swammerdam Institute for Life Science, University of Amsterdam, Amsterdam, Netherlands; ^5^Division of Periodontology, UConn Health, Farmington, CT, United States

**Keywords:** spores (bacterial), spore germination and inactivation (*B. subtilis*), spore membrane, spore resistance, spore killing

## Abstract

2Duf, named after the presence of a transmembrane (TM) Duf421 domain and a small Duf1657 domain in its sequence, is likely located in the inner membrane (IM) of spores in some *Bacillus* species carrying a transposon with an operon termed *spoVA*^2mob^. These spores are known for their extreme resistance to wet heat, and 2Duf is believed to be the primary contributor to this trait. In this study, we found that the absence of YetF or YdfS, both Duf421 domain-containing proteins and found only in wild-type (wt) *B. subtilis* spores with YetF more abundant, leads to decreased resistance to wet heat and agents that can damage spore core components. The IM phospholipid compositions and core water and calcium-dipicolinic acid levels of YetF-deficient spores are similar to those of wt spores, but the deficiency could be restored by ectopic insertion of *yetF*, and overexpression of YetF increased wt spore resistance to wet heat. In addition, *yetF* and *ydfS* spores have decreased germination rates as individuals and populations with germinant receptor-dependent germinants and increased sensitivity to wet heat during germination, potentially due to damage to IM proteins. These data are consistent with a model in which YetF, YdfS and their homologs modify IM structure to reduce IM permeability and stabilize IM proteins against wet heat damage. Multiple *yetF* homologs are also present in other spore forming *Bacilli* and *Clostridia,* and even some asporogenous *Firmicutes*, but fewer in asporogenous species. The crystal structure of a YetF tetramer lacking the TM helices has been reported and features two distinct globular subdomains in each monomer. Sequence alignment and structure prediction suggest this fold is likely shared by other Duf421-containing proteins, including 2Duf. We have also identified naturally occurring *2duf* homologs in some *Bacilli* and *Clostridia* species and in wt *Bacillus cereus* spores, but not in wt *B. subtilis*. Notably, the genomic organization around the *2duf* gene in most of these species is similar to that in *spoVA*^2mob^, suggesting that one of these species was the source of the genes on this operon in the extremely wet heat resistant spore formers.

## Introduction

Endospores formed by some *Bacilli* and *Clostridia* species are important vectors of human diseases and intoxications, and large amounts of food spoilage and food poisoning (Setlow and Johnson, 2019). These effects of spores are largely because of their extreme resistance that makes them difficult to kill by processes effective in killing growing cells (Christie and Setlow, 2021). A commonly used method for spore killing is wet heat, such as in a steam autoclave; indeed, spores or spore enzymes are commonly used indicators of efficient autoclave function (Vesley et al., 1992; Huesca-Espitia et al., 2016). In general, killing of well hydrated spores of *Bacilli* species requires temperatures ~40°C higher than those needed to kill growing cells (Warth, 1980).

Five factors contributing to spore wet heat resistance have been identified (Christie and Setlow, 2021). The first was spores’ accumulation of high levels, ~ 25% of spore core dry weight, of dipicolinic acid in a 1:1 chelate with Ca^2+^ (CaDPA) shown in the work of Joan Powell in the 1950s (Powell, 1953). The second factor was identified from work by Philip Gerhardt in the 1960s in collaboration with Robert Marquis, who showed that dormant spores’ core water content was as low as 25% of wet weight for a spore suspended in water but rose to ~80% of wet weight following completion of spore germination (Gerhardt and Marquis, 1989); notably, there is a good inverse correlation between spore core water content and wet heat resistance, with a lower core water content corresponding to a higher wet heat resistance. Indeed, one way in which CaDPA influences spore wet heat resistance is by its uptake into the spore core late in sporulation and displacement of some core water. For example, in *Bacillus subtilis* spores, CaDPA accumulation late in sporulation reduces spore core water content from 45 to 35% of wet weight and the DPA-less spores have significantly lower heat resistance than CaDPA replete spores (Paidhungat et al., 2000). The third factor was identified in the 1970s when the Setlow lab showed that endospores of all *Firmicute* species examined contain large amounts of small, acid-soluble proteins that saturate spore DNA and protect it from damage, including depurination and oxidation (Setlow, 1988). Spore DNA is thus so well protected against damage that spore killing by wet heat or an oxidative agent such as hydrogen peroxide (H_2_O_2_) is not by DNA damage, but by damage to some unidentified spore protein, although likely a protein essential for ATP generation early in the outgrowth of germinated spores (Setlow, 1988; Coleman et al., 2007; Coleman and Setlow, 2009; Korza et al., 2023). The fourth factor providing at least some of spores’ resistance to wet heat is the coat (Ghosh et al., 2009). The coat protection against wet heat may be due to the effects of the coat on spores’ inner membrane (IM) properties, as coat removal greatly increases spore IM permeability, even to water (Kaieda et al., 2013; Korza et al., 2023). The fifth factor was found a few years ago by a group at the University of Groningen in the Netherlands who identified and then sequenced the genomes of some extremely wet heat resistant *Bacillus* spores that had likely been selected for in a food processing plant in which wet heat treatment was used to inactivate spores in foodstuffs (Berendsen et al., 2016a,b; Luo et al., 2021). These very wet heat resistant spore formers had a transposable element with an operon termed *spoVA*^2mob^ transcribed only in the developing forespore in common, and the high spore wet heat resistance was largely due to one gene in the *spoVA*^2mob^ operon termed *2duf*. The encoded 2Duf protein consists of two domains of unknown function, an ~26 kD Duf421-like domain with an N-terminal transmembrane (TM) motif and an ~6 kD Duf1657-like domain at the C terminus; 2Duf is believed to be a spore IM protein. Notably, spores carrying the operon with *2duf* are resistant not only to wet heat but also to agents such as hydrogen peroxide (H_2_O_2_), nitrous acid and formaldehyde that must cross spores’ IM to damage one or more spore core or IM proteins (H_2_O_2_) or core DNA (formaldehyde, nitrous acid) (Kanaan et al., 2021; Korza et al., 2023). However, how 2Duf alters the IM to increase spore wet heat resistance and lower IM permeability is not understood.

The identification of a sixth factor important in spore resistance reported in this communication was somewhat fortunate, as the initial objective was to examine if the *yitF* gene into which the transposon noted above commonly integrated in *B. subtilis*, played any role in spore wet heat resistance. In beginning this experiment, weakening eyesight in the senior lab member read *yitF* as *yetF* and thus a *B. subtilis yetF* mutation was made as well an *ydfS* mutant. It turns out that YdfS and YetF are also Duf421-containing proteins but lack the C-terminal Duf1657 domain in 2Duf, although are homologs of ~80% of 2Duf. Notably, the *yetF* and *ydfS* genes are expressed in the developing spore, *ydfS* as a single gene recognized by the forespore-specific sigma G (σ^G^) subunit of RNA polymerase; *yetF* is reported as the second gene in a putative operon with *lplD* according to SubtiWiki but with its own σ^F^ promoter. However, *lplD* is a σ^E^-dependent gene expressed only in the mother cell compartment of the sporulating cell, and transcription of this gene likely contributes minimally to *yetF* expression (Arrieta-Ortiz et al., 2015). Importantly, as with 2Duf, YdfS and YetF are also predicted to be membrane proteins of relatively similar sequence and importantly, have been found in the *B. subtilis* spore IM (Zheng et al., 2016). It is thus tempting to speculate that YetF and YdfS may also play roles in spore heat resistance. Indeed, deletion of either *yetF* or *ydfS* decreased *B. subtilis* spores’ wet heat resistance markedly (see Results). All the current work following this observation now shows that YetF and its many homologs are new important factors in spore resistance and germination and has also revealed surprising information about the origin of 2Duf and the associated *spoVA*^2mob^ operon.

## Materials and methods

### Bacterial strains

Most *Bacillus* strains used in this work (Table 1) are isogenic with wild-type (wt) strain PS832, a prototrophic laboratory strain of *B. subtilis* 168, including PS533, also wt and containing plasmid pUB110 providing kanamycin resistance (Km^r^) (Fairhead et al., 1994). *B. subtilis* deletion-replacement mutants were constructed by transformation of strain PS832 with DNA of *B. subtilis* strains obtained from the *Bacillus* Genetic Stock Center (Koo et al., 2017); these strains have deletions of individual *B. subtilis* genes and their replacement by antibiotic resistance markers. Chromosomal DNA from such strains was used to construct *ydfS* deletions PS4481 and PS4483, and *yetF* deletions, PS4482 and PS4484, with the genes’ replacement by either a Km^r^ gene (PS4481, PS4482) or an erythromycin resistance gene (Em^r^) (PS4483, PS4484). PS4481 and PS4482 were then transformed to spectinomycin resistance (Sp^r^) with plasmid pDR244 (*Bacillus* Genetic Stock Center), inducing synthesis of the *cre* recombinase from the plasmid, selecting Sp^r^ transformants at 30°C and then at 42°C for loss of both the plasmid and Km^r^ (Koo et al., 2017). PCR analysis was then used to prove the *yetF* or *ydfS* genes were still absent, giving antibiotic resistance marker-free strains PS4487 (*ydfS*) and PS4488 (*yetF*). Additional strains were constructed in which the *yetF* gene’s own σ^F^ promoter or the very strong σ^G^-dependent promoter of the *sspA* gene (Arrieta-Ortiz et al., 2015) were cloned upstream of the *yetF* ribosome binding site and coding region in a modified pDG364 plasmid^1^ that allows stable integration of the plasmid encoded *yetF* genes at the *amyE* locus. These plasmids were used to transform PS832 and PS4488 to Km^r^, and the insertion of the genes and promoters at either *amyE* or *yetF* were verified by PCR and DNA sequencing, as was the case for other recombinant strains constructed in this work. Two additional strains isogenic only with each other are PS4461 (wt) and PS4462 (has *spoVA*^2mob^ with *2duf*) (Luo et al., 2021) and these were transformed to Km^r^ with chromosomal DNA from strain PS4482 thus deleting *yetF*, giving strains PS4489 from PS4461 and PS4490 from PS4462; the correct construction of these strains was also verified by PCR.

**Table 1:** *Bacillus subtilis* strains used in this work.

Isogenic strains	Relevant genotype	Source
PS832	Wild-type, prototrophic	Laboratory strain
PS533	PS832 plus pUB110, Km^r^	Fairhead et al. (1994)
PS4481	PS832 *ydfS* Km^r^	This work
PS4482	PS832 *yetF* Km^r^	This work
PS4483	PS832 *ydfS* Em^r^	This work
PS4484	PS832 *yetF* Em^r^	This work
PS4487	PS4481 *ydfS* Km^s^	This work
PS4488	PS4482 *yetF* Km^s^	This work
BH164	PS832 *PsspA-yetF* @*yetF* Km^r^	This work
BH165	PS832 *PyetF-yetF* @*amyE* Km^r^	This work
BH166	PS832 *PyetF-yetF @yetF* Km^r^	This work
BH167	PS4488 *PyetF-yetF @amyE* Km^r^	This work
Other isogenic strains		
PS4461	Wild-type	Luo et al. (2021)
PS4462	PS4461 with *spoVA*^2mob^	Luo et al. (2021)
PS4489	PS4461 *yetF*	This work
PS4490	PS4462 *yetF*	This work

Strains made in this work were constructed and constructions verified as described in Methods.

### Spore preparation and purification

Spores of various strains were prepared from log phase cells spread on 2xSG medium plates without antibiotics (Nicholson and Setlow, 1990; Paidhungat et al., 2000), plates incubated at 37°C for 2–3 d until sporulation was complete and spore release from sporangia was >95%. Spores were then scraped from plates into cold water, purified over 3–5 d by sonication and centrifugation with removal of supernatant fluid, and then centrifugation of spores through a high-density solution of 50% Histodenz in which spores pellet and debris floats (Setlow, 2019). Spores used in this work were > 98% free from germinated spores, growing or sporulating cells and debris as seen by phase contrast microscopy. In some cases, spores were chemically decoated by urea-SDS treatment as described previously (Korza et al., 2023).

### Spore properties

The wet density of spores of different strains was compared by centrifugation to equilibrium of 100 μL of spores at ~2×10^8^/mL in water layered on a ~ 2 mL 50–70% Histodenz gradient, with centrifugation for 45 min at 20°C at ~20,000 *g*, and spore’s banding positions in gradients were determined by inspection (Lindsay et al., 1985). The CaDPA content of 1.5 mL spores at an optical density at 600 nm (OD_600_) of 1.0 (~ 10^8^ spores/mL) was determined by boiling 1 mL in water for 30 min, cooling on ice, centrifugation in a microcentrifuge and determining relative CaDPA contents in the supernatant fluids by reaction with TbCl_3_ and measuring Tb^3+^-DPA fluorescence in a plate reader (Yi and Setlow, 2010). The precise number of spores used in each sample was determined by counting ~100 spores from each sample in a Petroff-Hausser Chamber (Luo et al., 2021). The amount of CaDPA relative to spore number was then calculated for spores of each strain.

Phospholipid analysis of vegetative cells was with cells grown at 37°C in LB medium to an OD_600_ of ~3, 35 mL of culture harvested by centrifugation, the pellet washed twice with 15 mL of sterile 0.15 M NaCl in 25 mM KPO_4_ buffer (pH 7.5), the pellet lyophilized, dry cells disrupted by bead beating, phospholipids extracted as described previously and the extracts dried (Korza et al., 2023). Dormant spores (~40 mg dry weight) were decoated to remove outer membrane lipids and adventitiously adsorbed lipids, washed, spores disrupted in a bead beater, phospholipids extracted, extracts dried, all dry extracts dissolved in a small volume of methanol and analyzed in duplicate by mass spectrometry as described (Korza et al., 2023).

To assess rates of spore germination, spores at an OD_600_ of 1.0 were heat activated at 80°C for various times and cooled on ice. Spores with heat activation times giving maximum germination rates were germinated at 37°C and an OD_600_ of 0.5 in 200 μL of 25 mM K-Hepes buffer (pH 7.4) and 50 μM TbCl_3_, and either 10 mM L-valine or the AGFK germinant mixture (10 mM each of L-asparagine, D-glucose, D-fructose and KCl), and CaDPA release was monitored in a fluorescence plate reader as described (Yi and Setlow, 2010). Spore germination in 1 mM dodecylamine without heat activation used spores at an OD_600_ of 0.5 at 45°C in 25 mM K-Hepes buffer pH 7.4; 190 μL samples taken at various times were added to 10 μL of 1 mM TbCl_3_ and fluorescence was measured in a plate reader.

For measurement of germination parameters of individual PS832 (wt) and PS4488 (*yetF*) spores, spores in water to be germinated with L-valine were heat activated at 80°C for 60 min (PS832) or 30 min (PS4488), and for germination with 10 mM of each AGFK component for 75 min (PS832) or 45 min (PS4488). After cooling, germination of multiple individual spores at 37°C in 25 mM K-Hepes buffer, pH 7.4 was followed by phase contrast microscopy as described previously (Kong et al., 2011, 2014). Briefly, spores were spread on the surface of a microscope coverslip that was dried in a vacuum desiccator for 10–20 min. Coverslips were then mounted on and sealed to a microscope sample holder kept at room temperature. After adding either the L-valine or AGFK buffer mixture to spores on the coverslips, a digital CCD camera (12 bit, 1,340 by 1,024 pixels) was used to record the images at a rate of 1 frame per 15 s for 60–120 min with ~300 individual spores. These images were analyzed with a computation program in Matlab to locate each spore’s position and to calculate the averaged pixel intensity of an area of 20 × 20 pixels that covered the whole individual spore’s phase contrast image. The phase contrast image intensity of each individual spore was plotted as a function of the incubation time (with a resolution of 15 s) and the intensity at T0 (the first phase contrast image recorded after the addition of the germinant) was normalized to 1 and the intensity at the end of measurements was normalized to zero. This analysis allowed the determination of kinetic germination parameters of multiple individual spores, including the time T*_lag_* before rapid CaDPA release began, although there is some CaDPA leakage during this period; T_release_, the time when CaDPA release is complete; and T*_lys_*, the time for completion of cortex lysis (see Results).

Determination of rates of permeation of a molecule across the IM and into the core of intact or urea-SDS decoated spores, as well as core pH, was by measuring ^14^C-methylamine incorporation (Cortezzo et al., 2004; Korza et al., 2023). Spores (~15 mg dry weight/mL) in 200 mM Tris–HCl (pH 8.8) were incubated at 23°C with 5 mM ^14^C-methylamine (Moravek Biochemicals, Brea, CA, United States) (~ 2.5×10^5^ dpm/mL). At various times, 600 μL samples were rapidly passed through a 0.22-micron centrifuge filter, the filtrate saved, the filter washed with two ~600 μL aliquots of 4°C 200 mM Tris–HCL buffer (pH 8.8), and the filtrates saved. All filtrates were made 50 g/L in trichloroacetic acid (TCA), and 600 μL of 50 g/L TCA was added above the filter. After incubation overnight to elute ^14^C-methylamine from spores, the filter was centrifuged to collect the eluate, and 500 μL of the eluate and 500 μL of the initial filtrate and washes were added individually to 4 mL scintillation fluid and samples were counted in a scintillation counter.

### Spore killing by wet heat and other agents

Spores at an OD_600_ of 1 in water were incubated at 90–98°C and at various times 100 μL samples were diluted 1/10 in cold sterile water. Different temperatures were chosen in different experiments for a variety of reasons including: (i) maximally emphasizing differences seen in pilot experiments, and ensuring that effects seen were maximal at certain conditions and (ii) fitting experiments into time schedules of students. This 1/10 dilution was serially diluted 10-fold to 1/10^5^, and 10 μL aliquots were spotted in duplicate on LB medium plates with only one antibiotic if applicable. After liquid soaked in, the plates were incubated overnight at 30°C and then at 37°C until no more colonies appeared and colonies were counted. All heat resistance experiments were carried out with duplicate determinations at each time point, such that all data points shown are averages of at least two measurements for each value; in general, individual values differed by ≤30% and ≤ 25% for spore killing ≤99%. All killing experiments were carried out at least twice with essentially identical results.

Spore killing by H_2_O_2_ was at 23°C in 25 mM K-Hepes buffer (pH 7.0) with 11% H_2_O_2_ and spores at an OD_600_ of 1 (~ 10^8^ spores/mL). At various times aliquots (100 μL) were diluted 1/10 into a catalase solution to inactivate H_2_O_2_ as described (Kanaan et al., 2021), this sample diluted further, aliquots of dilutions spotted in duplicate on plates, plates incubated, and colonies were counted as described above. Spore killing by 2.5% formaldehyde in water at 30°C and by 400 mM NaNO_2_ in 200 mM acetate buffer (pH 4.5) and 30°C were carried out as described previously, including inactivating the sporicidal agent to preventing further spore killing (Kanaan et al., 2021). Spore killing by octanol or chloroform was determined using spores at an OD_600_ of 2 in 23°C water with 10% octanol or neat chloroform, the suspension vortexed intermittently over 15 min, and aliquots were applied to a grid on LB plates as described above to assess spore viability. Assessment of spore lysozyme resistance was by incubating spores at an OD_600_ of 1 in 25 mM Tris–HCl buffer (pH 8.0) with or without 15 μg/mL lysozyme, incubating for 60 min at 37°C and examining the spores by phase contrast microscopy to identify spores that had germinated or germinated and lysed.

### Blast search, sequence alignment and computed 3D structural models

The search for homologs of *B. subtilis* YetF was conducted using its amino acid sequence (GenBank accession number NP_388595) as a query on the SubtiWiki server.^2^ The search included completed genomes of a variety of *Bacilli* and *Clostridia* species, both spore forming and asporogenous species, though not all species were screened. Hits were selected based on E-value < e^−30^ and query coverage >85%. The identified hits were then manually grouped into two categories based on amino acid residue numbers close to that of YetF or 2Duf, with the best hit for each species selected based on the highest E-value. The numbers of YetF/2Duf homologs found in each species are summarized in Table 2 and Supplementary Tables S2, S3. ClustalW alignments of best YetF or 2Duf homologs from *Bacilli* and *Clostridia* were performed using DNASTAR lasergene suite 8 (DNASTAR Inc.) with the default settings. Computed structural models for full-length YetF and 2Duf were generated using AlphaFold (Jumper et al., 2021). Five models were calculated for each protein sequence and only the top-ranked model was presented in figures. Molecular graphics were rendered using PyMol (Schrödinger LLC).

**Table 2:** Sequence conservation of YetF and 2Duf homologs in *B. subtilis* and other *Firmicutes.*

Species	Homologs (numbers of species)	Amino acids and range	Sequence identity or range (%)	
			YetF	2Duf
*B. subtilis*	2Duf	286	31.5	–
	YetF	231	–	31.5
	YdfS	235	35.8	31.2
	YkjA	243	23.8	26.8
	YrbG	218	23.5	30.4
	YdfR	225	21.9	26.9
*Bacilli*	YetF-like (36)	218–240	22.1–73.4	–
	2Duf -like(20)	285–290	–	33.9–60.8
*Clostridia*	YetF (37)	210–253	24.3–34.6	–
	Likely *spo*^+^ YetF-like (20)	225–250	24.9–34.4	–
	2Du-like (6*)	285–286	–	38.6–54.0

*None in the Likely *spo*^+^.

## Results

### Identification YetF homologs, and effects of *yetF* and *ydfS* mutations on spore resistance

**Figure 1:**
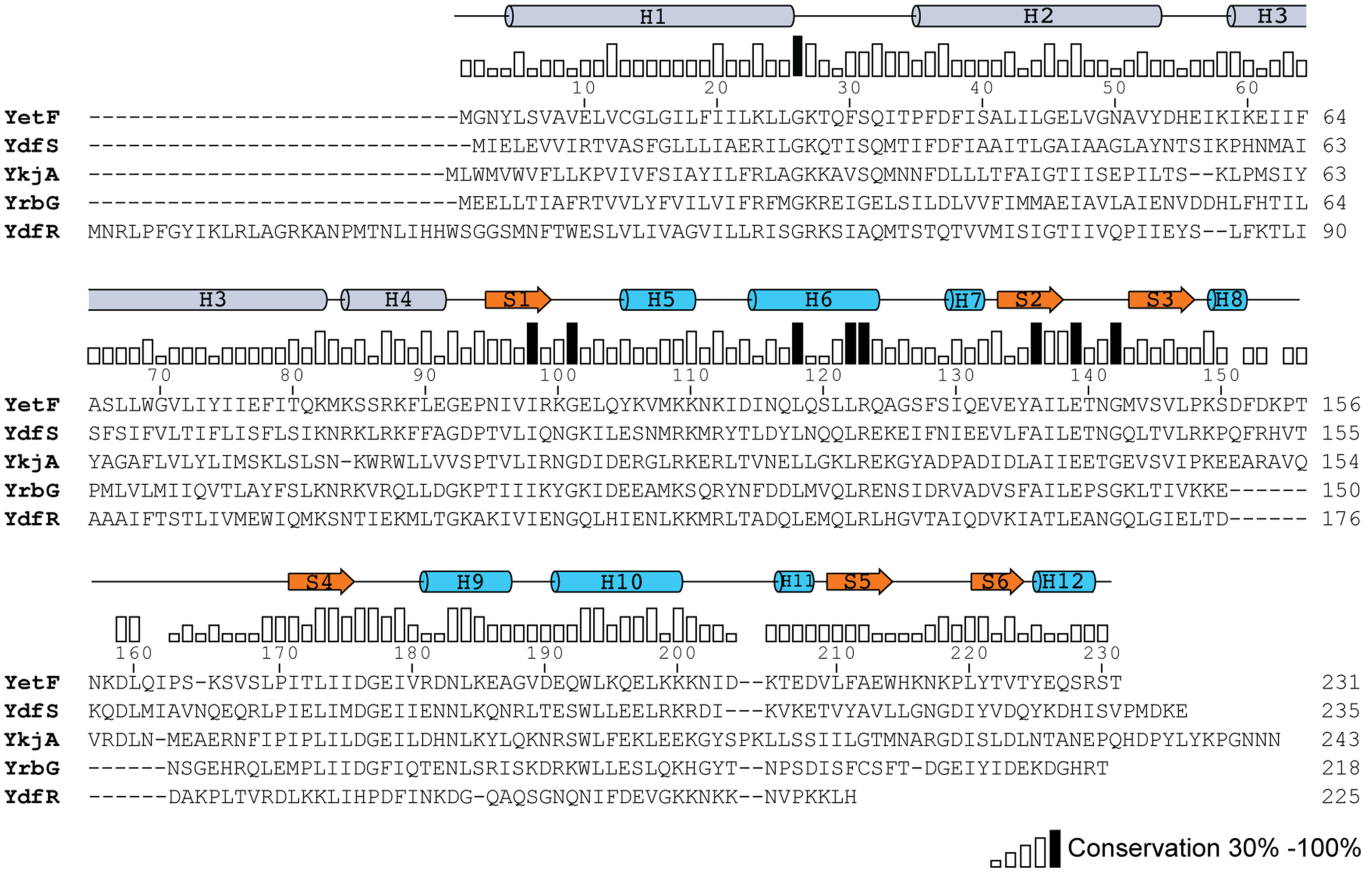
Sequence conservation between YetF and its homologs in *B. subtilis*. The YetF homologs were identified in a Blast search of the *B. subtilis* genome against the YetF sequence. Sequence conservation is shown as a bar graph, with black bars indicating identity among five proteins. Secondary-structure assignments of YetF from the crystal structure (PDB ID: 3C6F) are shown as blue cylinders (helices) and orange arrows (β strands). Predicted secondary-structure elements by AlphaFold for the N-terminal TM region are shown as gray cylinders (α helices).

Blast search using YetF as the query sequence identified four YetF homologs (YdfS, YkjA, YrbG, and YdfR) in the *B. subtilis* 168 reference genome that lacks the *spoVA*^2mob^ operon (Table 2 and Figure 1). Pairwise sequence alignment of these homologs showed that they share a sequence identity of 21.9 to 35.8% with YetF, and only contain a Duf421-like domain with amino acid residues ranging from 218 in YrbG to 243 in YkjA. Importantly, despite the absence of the Duf1657 domain, YetF and its homologs share 26.9 to 31.5% pairwise sequence identity with 2Duf (Table 1), indicating a likely functional similarity between YetF and 2Duf. Therefore, in subsequent studies, we focused on *yetF* and *ydfS* genes as: (i) the encoded proteins were most similar to the Duf421 domain in 2Duf; and (ii) YetF is the most abundant of the five homologs in spores, and YdfS is one of the two that are least abundant (Zheng et al., 2016).

**Figure 2:**
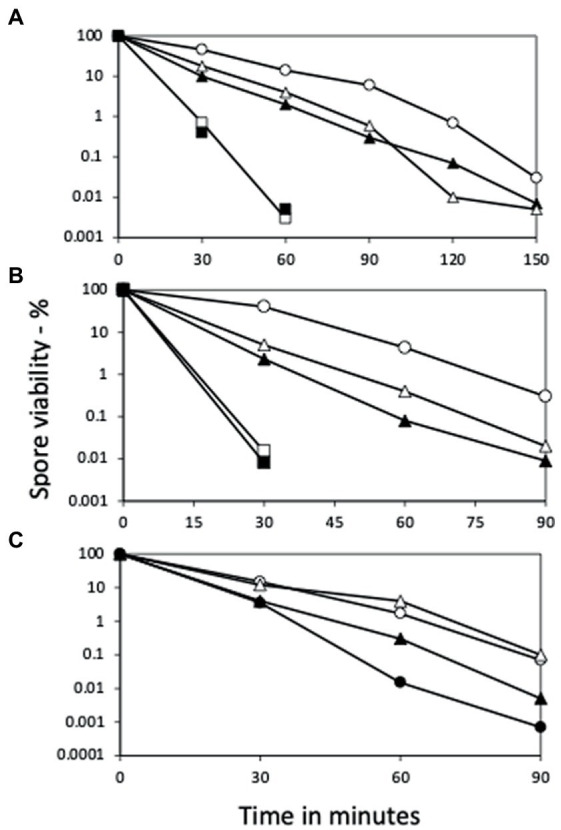
Wet heat killing of spores of various strains with and without *ydfS* and *yetF* mutations, and with and without antibiotic markers. Spores of *B. subtilis* strains were incubated in water at **(A)** 90°C or **(B)** 93°C or **(C)** 93 or 98°C for various times, samples diluted 1/10 in cold water, then diluted serially 1/10-fold, 10 μL aliquots spotted on LB plates without antibiotics, incubated at 30°C and then 37°C until no more colonies appeared, and colonies were counted, all as described in Methods. The symbols for the spores used in the figures are: **(A)** ○ – PS533 (wt), ▲ – PS4481 (*ydfS* Km^r^), □ – PS4482 (*yetF* Km^r^), is △ – PS4483 (*ydfS* Em^r^), and ■ – PS4484 (*yetF* Em^r^); **(B)** ○ – PS832 (wt), △ – PS4481 (*ydfS* Km^r^), □ – PS4482 (*yetF* Km^r^), ▲ – PS4487 (*ydfS*), and ■ – PS4488 (*yetF*); and **(C)** at 93°C ○ – PS4461 (wt); ● – PS4489 (PS4461 *yetF*), and at 98°C with △ – PS4462, and ▲ – PS4490 (PS4462 *yetF*). Data are averages from three separate experiments and results varied by ≤22%.

Initial results examining the heat resistance of *B. subtilis yetF* and *yfdS* spores used mutants that had antibiotic resistance genes replacing coding genes (Figure 2A), and either mutation resulted in decreased spore wet heat resistance, with *yetF* spores having lower resistance than *ydfS* spores. To ensure that the effects of the mutations were not influenced by the antibiotic resistance genes introduced in mutant generation, the antibiotic resistance markers were removed from one set of strains (Km^S^). However, the heat resistance of the spores without the antibiotic resistance genes were essentially identical to those of spores that retained this marker (Figures 2A,B); note that the temperatures used in panels A and B, were 90 and 93°C, respectively.

Given that the presence of 2Duf greatly increases spore wet heat resistance (Luo et al., 2021), an obvious question is whether deletion of *yetF* in spores with 2Duf will decrease these spores’ wet heat resistance. Indeed, when the *yetF* mutation was introduced into isogenic strains PS4461 (wt) and PS4462 (with *2duf*), spores of both strains lacking *yetF* were less heat resistant than spores of their parents (Figure 2C); note in this experiment that spores of the PS4462 strains were incubated at 98°C, while spores of the PS4461 strains were incubated at 93°C.

**Figure 3:**
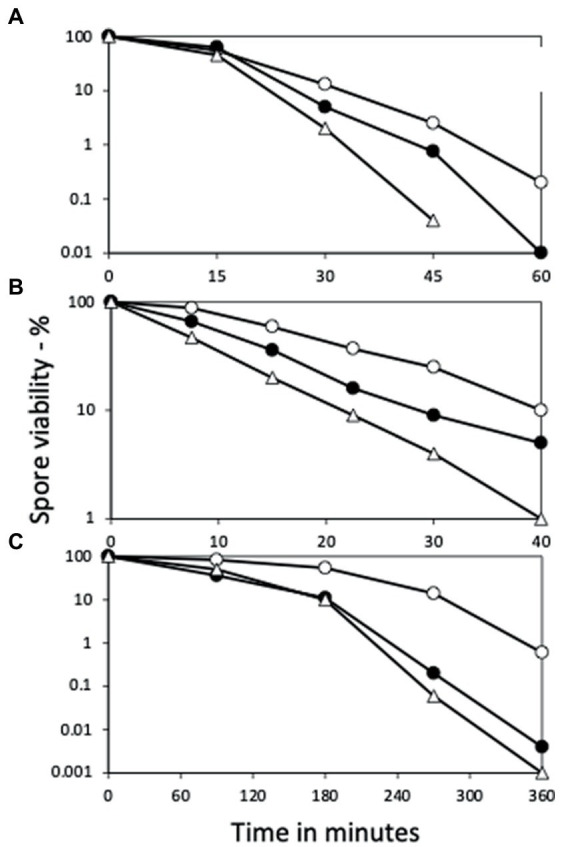
Effects of *yetF* and *ydfS* mutations on additional spore resistance properties. Spores of strains PS832 (wt), PS4487 (*ydfS*), and 4488 (*yetF*) were treated with **(A)** H_2_O_2_, **(B)** formaldehyde, or **(C)** nitrous acid, and spore survival was measured, all as described in Methods. The symbols used are: ○ – PS832, ● – PS4487 and △ – PS4488. Data are averages from three independent experiments, results varied by ≤20%.

**Figure 4:**
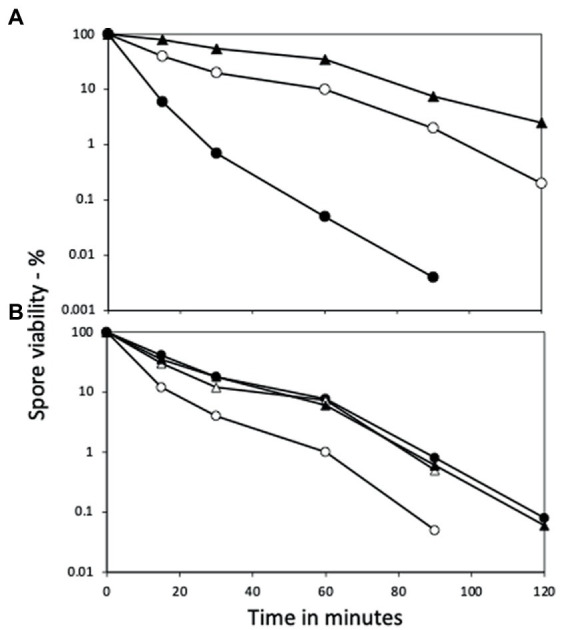
Wet heat resistance of spores of wt and *yetF* strains with *yetF* at different loci and under control of different promoters. **(A)** Spores of PS832 (wt), PS4488 (*yetF*) and BH167 (PS4488 with *yetF* at *amyE* under control of the *yetF* promoter) and **(B)** PS832, BH164 (PS832 with *yetF* at the *yetF* locus under control of the *sspA* promoter), BH165 (PS832 with *yetF* at *amyE* under control of the *yetF* promoter) and BH166 (PS832 with *yetF* at the *yetF* locus under control of the *yetF* promoter) were wet heat treated at either **(A)** 90°C or **(B)** 95°C and spore viabilities were determined as described in Methods. Symbols used are: **(A)** ○ – PS832, ● – PS4488, and ▲ – BH167; and **(B)** ○ – PS832, ● – BH164, △ – BH165, and ▲ – BH166. Results are from two experiments where duplicates varied by ≤30%.

Consistent with the role of YetF and YdfS in spore heat resistance and their close relationship with 2Duf, *yetF* and *ydfS* genes exhibit the highest expression late in sporulation (see transcription browser in SubtiWiki; Pedreira et al., 2022) and their gene products have been identified in the IM fraction of spores of *B. subtilis* and homologs in *Bacillus cereus* spores’ IM (Zheng et al., 2016; Gao et al., 2021). Since perturbation of spores’ IM has been shown to alter spore resistance not only to wet heat but also to agents that must cross the IM to damage spore core components, including DNA (formaldehyde and nitrous acid) or a core protein (H_2_O_2_) (Kanaan et al., 2021), the effects of *yetF* and *ydfS* mutations on spore resistance to these agents was also examined (Figures 3A–C). Again, the mutations decreased spore resistance to these agents, the *yetF* mutation more so. While the *yetF* mutation had a large effect on spore resistance, it was important to show that these effects, in particular on spore wet heat resistance, could be reversed by complementation with the appropriate wt gene. This was achieved by cloning *yetF* in an integrative plasmid under control of its own σ^F^ promoter expressing *yetF* alone and using the plasmid with the appropriate construct to transform PS4488 by integration at *amyE*. Notably, the wet heat resistance of spores of the *yetF* mutant was restored close to wt spore resistance by *yetF* insertion at *amyE* (Figure 4A). This latter result also indicates that the *lplD* gene preceding *yetF* operon likely plays no role in YetF’s effects on spore wet heat resistance. This same plasmid, as well as the comparable plasmid with *yetF* under the control of the strong forespore-specific *sspA* promoter were also transformed into the wt strain PS832, and integrants at both *amyE* and the *yetF* loci were identified. Notably, expression of *yetF* at the *yetF* locus from the strong forespore-specific *sspA* promoter as well as from the *yetF* promoter, or at either *yetF* or *amyE* from the *yetF* promoter gave spores with higher wet heat resistance than wt spores (Figure 4B). That expression of *yetF* from the *sspA* promoter and *yetF* expression from its own promoter (BH164, BH165) gave no higher spore wet heat resistance than with expression of two *yetF* genes from the *yetF* promoter (BH166) was somewhat surprising given how strong the *sspA* promoter is such that SspA is one of the most abundant spore proteins. However, we do not know how much YetF was accumulated in the spores with *yetF* under the different promoters, how much YetF accumulation alone can increase spore wet heat resistance, nor how YetF levels may interact with or influence other factors involved in spore wet heat resistance, such as coat structure and even spore DNA structure.

In contrast to the effects of *yetF* and *ydfS* mutations on spore resistance noted above, treatment of these mutant spores with chloroform or octanol gave no detectable spore killing (data not shown). In addition, incubation of PS832, *yetF* or *ydfS* spores with lysozyme as described in Methods resulted in ≤2% germination for all three types of spores (data not shown). These negative results indicate that YetF and its homologs do not exert their effects by altering spore coat structure, as an intact spore coat is essential in spores’ resistance to octanol, chloroform, or lysozyme. The wt, *yetF* and *ydfS* strains also grew at essentially the same rates in LB liquid medium when inoculated as vegetative cells, and the spores of these strains germinated, outgrew and then grew vegetatively in LB medium supplemented with L-valine at very similar rates starting from spores (Supplementary Figure S1).

### Core water, CaDPA and phospholipid levels in spores of strains lacking YetF or YdfS

While the results noted above suggest that *yetF* and *ydfS* mutations exert their effects by altering spore IM properties, perhaps IM permeability and/or rigidity, other mechanisms are certainly possible. Two such alternatives are altering spore core water content and/or CaDPA levels (Gerhardt and Marquis, 1989). However, when the core water and CaDPA contents of spores of strains PS832, PS4487 and PS4488 were determined (Table 3), the analyses showed that spores of these strains had essentially identical CaDPA and core water contents. Thus, changes in the amounts of these spore components play no role in the effects of *yetF* and *ydfS* mutations on spore resistance.

**Table 3:** Levels of CaDPA and core water content in spores +/− YetF or YdfS.

Spores examined	PS832 (wt)	PS4487 (*ydfS*)	PS4488 (*yetF*)
Relative CaDPA Content^1^	100	92	97
Relative core wet density^2^	64^3^	65	65

Spores were prepared and purified and relative levels of CaDPA in spores, and the banding position of spores in a 50–70% Histodenz gradient, a reflection of spore core water content, were determined as described in Methods.

1 Value set at 100 for wt spores.

2 The number given is the position where the spores banded in a Histodenz gradient, as the % Histodenz at this position.

3 These spores gave a significantly wider band than PS4487 and PS4488 spores.

Another possible cause for the different resistance properties of *ydfS* and *yetF* spores compared to spores of their wt parent could be that the mutant spores have different levels of specific IM phospholipids. Indeed, previous work showed that drastic alterations in spore IM phospholipid composition, such as the absence of phosphatidylethanolamine (PE) or cardiolipin (CL) alone increased rates of spore killing by wet heat 2- and 10-fold, respectively, although rates of H_2_O_2_ killing by at most 2-fold (Griffiths and Setlow, 2009). However, recent work showed that spores with and without 2Duf had almost identical IM phospholipid levels despite the much higher wet heat resistance of spores with 2Duf (Korza et al., 2023). Analysis of IM phospholipid levels in wt, *yetF* and *ydfS* growing cells and spores found higher levels of phosphatidylglycerol and CL and lower levels of PE and unknown phospholipids in spores compared to vegetative cells (Table 4). However, PE levels were only ~20% lower in *yetF* or *ydfS* spores compared to wt spore levels, and CL levels were actually ~20% higher in *yetF* and *ydfS* spores (Table 4). Thus the magnitude of these differences in IM phospholipid levels seem very unlikely to be a major factor in the decreased resistance of *yetF* and *ydfS* spores (and see below).

**Table 4:** Phospholipid levels in spores and vegetative cells of different strains.

	Phospholipid levels – % of total +/− SD			
Strain	PG	CL	PE	UNK
	Spores			
PS832 – wt	83.4 ± 2.9	9.8 ± 0.2	4.8 ± 0.1	1.9 ± 0.4
PS4487 – *ydfS*	82.0 ± 8.5	12.0 ± 0.3	3.7 ± 0.1	1.3 ± 0.1
PS4488 – *yetF*	80.6 ± 8.0	13.6 ± 0.8	3.7 ± 0.4	2.1 ± 0.4
	Vegetative cells			
PS832 – wt	69.3 ± 1.1	5.7 ± 0.1	13.4 ± 0.8	11.4 ± 0.3
PS4487 – ydfS	69.2 ± 1.6	5.7 ± 0.1	12.8 ± 0.3	11.4 ± 0.2
PS4488 – yetF	69.0 ± 2.6	5.8 ± 0.6	12.8 ± 0.6	11.7 ± 0.6

Phospholipids were extracted from dormant spores or vegetative cells and analyzed by mass spectrometry as described in Methods. Values for spores and vegetative cells are from duplicate analyses +/− standard deviations (SD). Abbreviations for phospholipids are: PG – phosphatidylglycerol, CL – cardiolipin; PE – phosphatidylethanolamine; and UNK – unknown.

### Effects of *yetF* and *ydfS* mutations on spore germination, IM germination proteins and permeability

**Figure 5:**
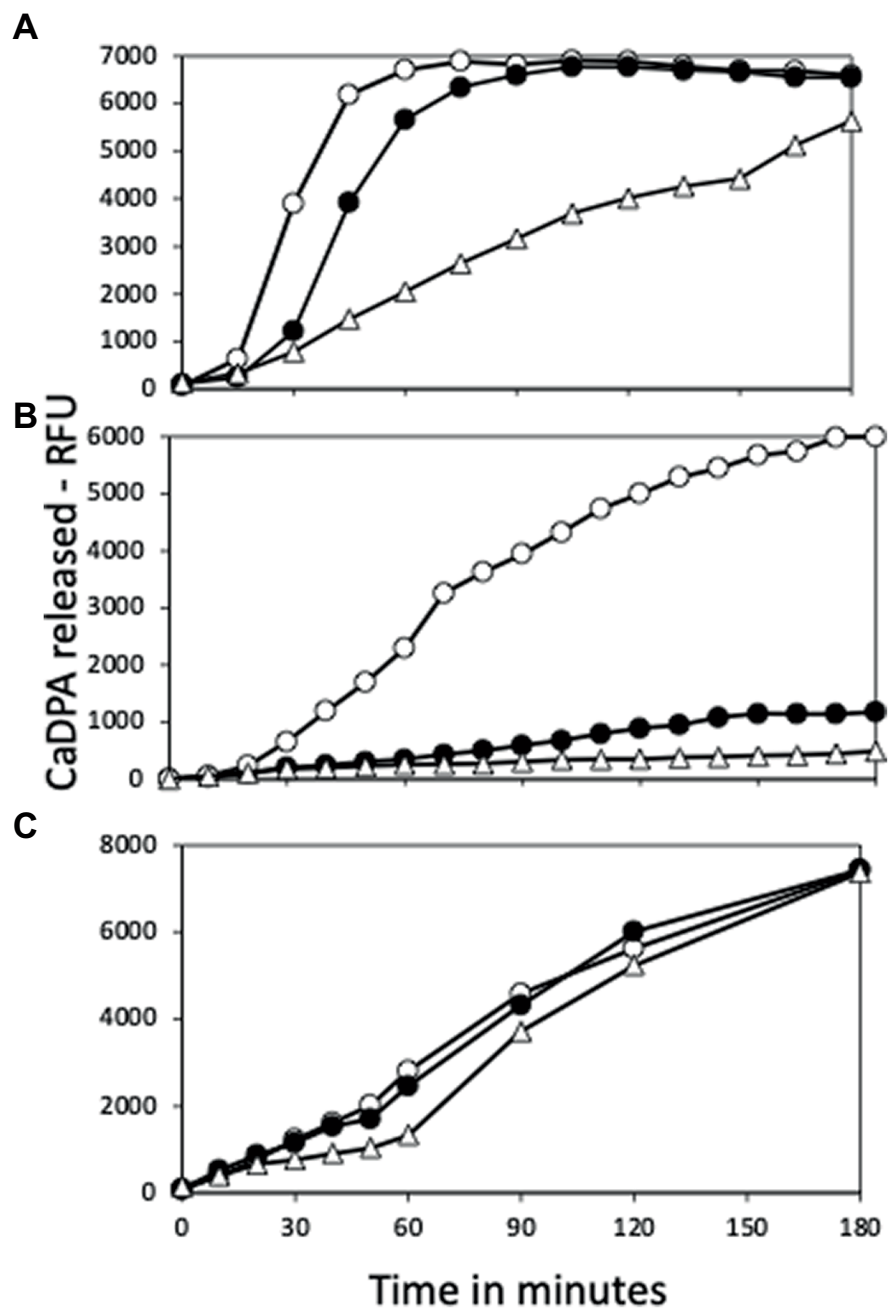
Germination of spores with or without *yetF* or *ydfS* with **(A)** L-valine, **(B)** AGFK or **(C)** dodecylamine. Spores of strains PS832 (wt – ○), PS4487 (*ydfS* – ●), and PS4488 (*yetF* – △) were optimally heat activated at 80°C **(A,B)**, cooled, and germinated with **(A)** L-valine, **(B)** AGFK or **(C)** dodecylamine as described in Methods, and spore germination was measured by release of CaDPA and expressed as relative fluorescence units (RFU). Values in **(A,B)** are averages of duplicate determinations, while values in **(C)** are from single measurements. Spores of all three strains used in this experiment had essentially identical amounts of CaDPA (data not shown). Values in **(A,B)** are from samples given the optimum heat activation times for germination with L-valine (Figures 6A–C) which were: **(A)** – 105 min, **(B)** – 60 min, and **(C)** – 30 min; or with AGFK (Figures 7A–C) were **(A)** – 75 min, **(B)** – 120 min, and **(C)** – 30-120 min. Values are from two measurements in two independent experiments.

**Figure 6:**
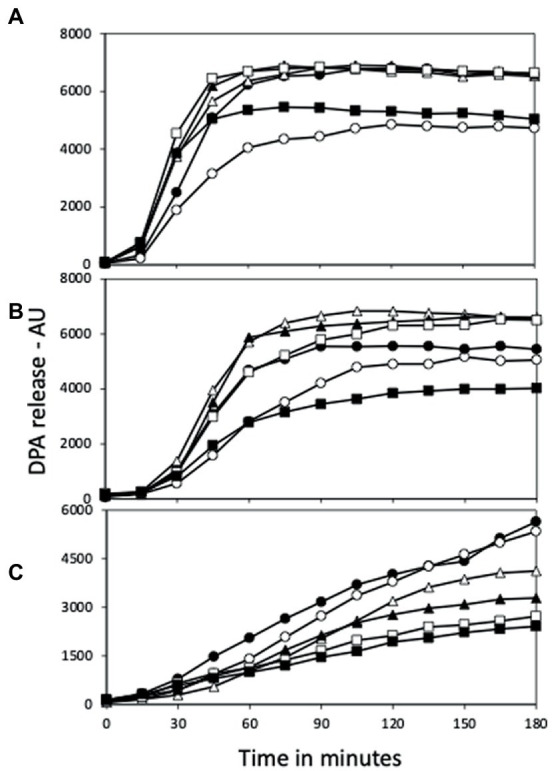
L-Valine germination of **(A)** PS832 (wt), **(B)** PS4487 (*ydfS*) and **(C)** PS4488 (*yetF*) spores after various heat activation times. Spores of various strains at an OD_600_ of 1 were incubated in water at 80°C, and at various times aliquots were removed and cooled and germinated with L-valine and germination followed in a plate reader measuring fluorescence of released DPA in arbitrary units (AU) as described in Methods. All values shown are averages of duplicate determinations in two experiments. The symbols for the various heating times are: 0 min – ○; 30 min – ●; 60 min – △; 90 min – ▲; 120 min – □; and 150 min – ■.

**Figure 7:**
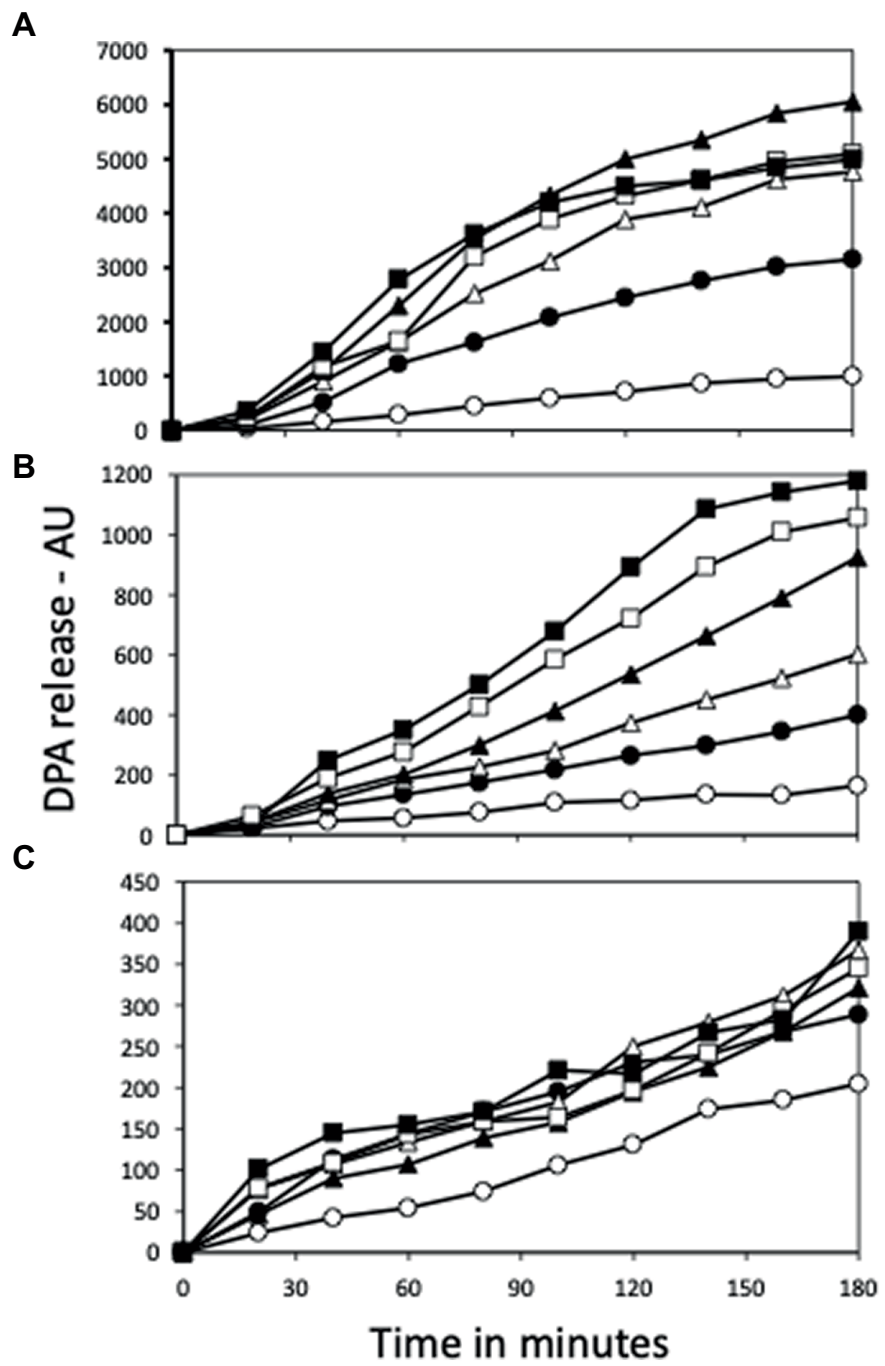
AGFK germination of **(A)** PS832 (wt), **(B)** PS4487 (*ydfS*) and **(C)** PS4488 (*yetF*) spores after various heat activation times. Spores of various strains at an OD_600_ of 1 were incubated in water at 80°C, and at various times aliquots were removed and cooled and then germinated with AGFK and germination followed in a plate reader measuring fluorescence of released DPA in AU as described in Methods. All values shown are averages of duplicate determinations. The symbols for the various heating times are: 0 min – ○; 15 min – ●; 30 min – △; 60 min – ▲; 90 min – □; and 120 min – ■.

Since YetF and YdfS are in spores’ IM and alter spores’ resistance to agents that cross the IM to damage core components, possibly these proteins modify IM structure and properties in some fashion. In turn, this might alter properties of IM proteins, some of which are crucial in spore germination. Indeed, recent work has shown that the presence of the likely IM protein 2Duf, to which YetF and YdfS exhibit partial homology, stabilizes IM germination proteins against wet heat, and makes the GerB and GerK germinant receptors (GRs) more resistant to heat activation for subsequent spore germination (Krawzyck et al., 2017; Korza et al., 2023). Consequently, the germination of wt, *yetF* and *ydfS* spores *via* the GR GerA recognizing L-valine, the GerB and GerK GRs together responding to the AGFK mixture, or with dodecylamine which is not a GR-dependent germinant, were examined, measuring CaDPA release to monitor germination (Figures 5A–C). Using spore heat activation times producing the fastest L-valine germination (Figures 6A–C), L-valine germination of *yetF* spores was significantly slower than that of wild-type spores, *ydfS* spore germination with L-valine was slightly slowed (Figure 5A), and germination with the AGFK mixture of spores given heat activation promoting the fastest AGFK germination (Figures 7A–C) was almost abolished by the *yetF* mutation and greatly slowed in *ydfS* spores (Figure 5B). However, the wild-type and mutant spores exhibited similar rates of dodecylamine germination (Figure 5C). Note that the absence of either PE or CL from spores only decreased rates of spore germination at most ~2-fold (Griffiths and Setlow, 2009). These results are thus further evidence that effects of *yetF* or *ydfS* on spore properties are not the result of a drastic change in IM phospholipid composition in these mutant spores.

**Figure 8:**
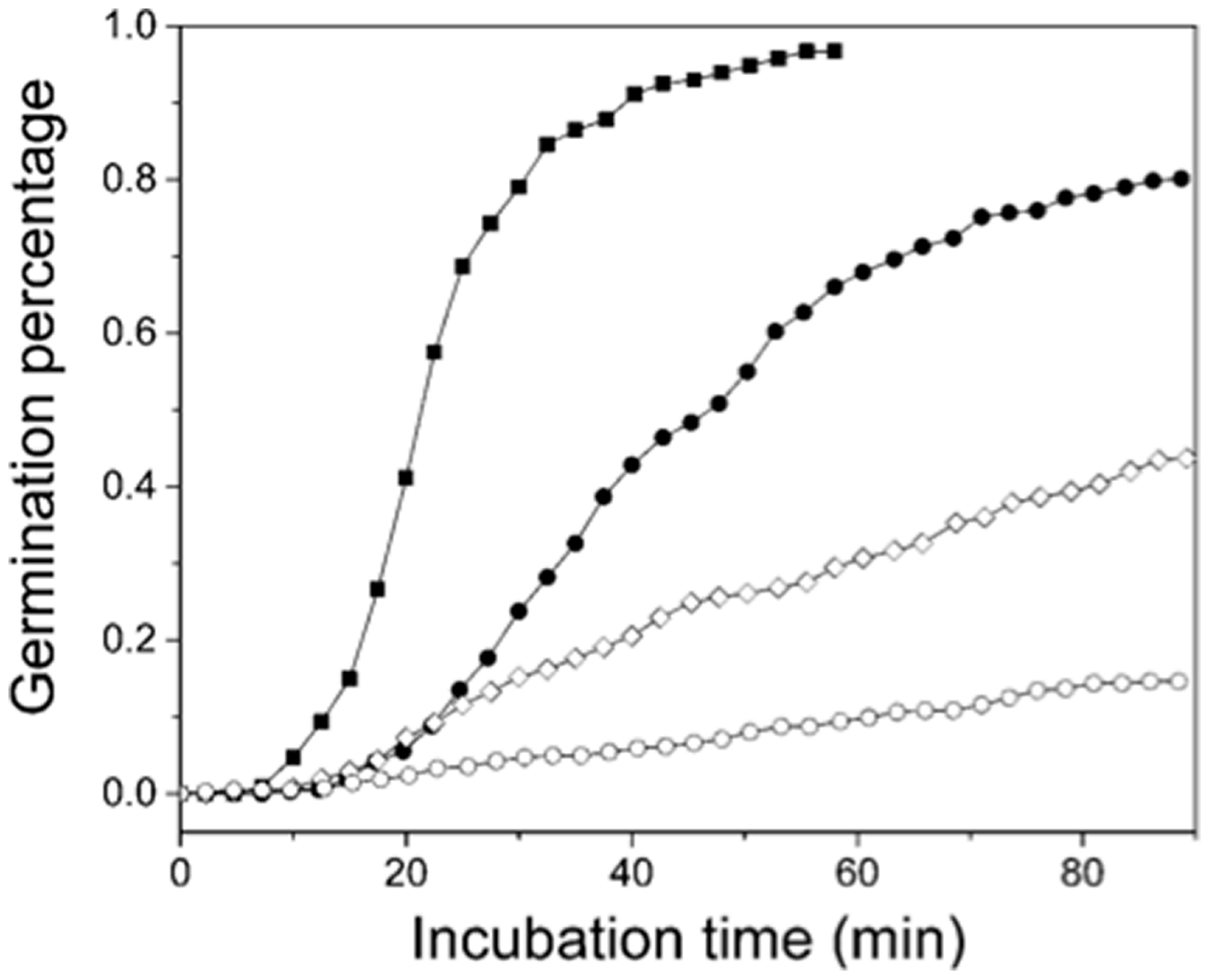
Germination of multiple individual PS832 (wt) and PS4488 (*yetF*) spores with L-valine or AGFK in 25 mM Hepes (pH 7.4) at 37°C. The symbols for the spores and germinants used are: PS832 spores germinated with L-valine (■) or AGFK (●); and PS4488 spores germinated with L-valine (◇) or AGFK (○). The germination of ~300 individual spores was followed as described in Methods.

The germination experiments discussed above were carried out with spore populations, and studies have shown there is significant heterogeneity between individual spores in their germination, and that additional information can be obtained by examining the kinetic parameters of the germination of multiple individual spores (Setlow et al., 2017). Consequently, the germination of large numbers of individual spores of PS832 and PS4488 spores germinating with L-valine or AGFK were followed, and decreased rates of L-valine and AGFK germination of PS4488 spores were again seen (Figure 8). Following germination of multiple spores individually (Supplementary Figures S2A,B) also allowed determination of the various average kinetic parameters of spore germination (Supplementary Table S1), including T*_lag_*, T*_release_* and T*_lys_*, as well as ΔT*_release_*, the time for rapid CaDPA release, T*_lag_* – T*_release_*; ΔT*_lys_*, the time for cortex lysis, T*_lys_* – T*_release_*; and the average spore image intensities at T*_lag_* (I*_lag_*) and T*_release_* (I*_release_*) (Supplementary Figures S2A,B). One notable value is the I*_lag_* which gives an estimate of how much CaDPA has leaked out during the T*_lag_* period, and the lower average I*_lag_* value for *yetF* spores with both germinants is consistent with these spores’ longer average T*_lag_* times. Another notable difference in germination parameters of wt and *yetF* spores was longer ΔT*_lys_* times for *yetF* spores, while ΔT*_release_* times were relatively similar with both strain’s spores.

The effects of different heat activation times on rates of L-valine germination of the spores of the wt, *yetF* and *ydfS* strains (Figures 6A–C) were also notable, as the rate of germination decreased after 120 min at 80°C with wt spores (Figure 6A), after 60 min with *ydfS* spores (Figure 6B) and after 30 min with *yetF* spores (Figure 6C). However, the maximum CaDPA release by the L-valine-germinating spores of the three strains in this experiment were essentially identical. These findings suggest that compared to wt spores, proteins essential for CaDPA release in L-valine germination are less resistant to wet heat in *ydfS* spores and even less resistant in *yetF* spores. The situation with AGFK germination was complicated by the fact that the *ydfS* and the *yetF* mutations decreased the maximum CaDPA release to ~20 and 6%, respectively, of that for wt spores. However, the large decreases in AGFK germination in the *yetF* and *ydfS* spores during heat activation (Figures 7A–C) are consistent with lower wet heat resistance of IM proteins needed for AGFK germination in these spores compared to their resistance in wt spores. Despite the differences in spore germination rates and extents noted above, the wt, *yetF* and *ydfS* spores germinated, outgrew and also grew vegetatively in the rich LB medium supplemented with L-valine at very similar rates starting from spores (Supplementary Figure S1).

Another important IM property is its permeability to small uncharged molecules, as this is very low across the spore IM relative to that across the germinated spores’ plasma membrane which is derived from the IM. The lower resistance of *yetF* and *ydfS* spores to agents that must cross the IM to damage core components as noted above suggests that loss of these proteins increases spore IM permeability. Consequently, uptake of ^14^C-methylamine by both intact and decoated PS832, PS4487 and PS4888 spores was measured (Supplementary Figures S3A,B). However, there were no significant differences in the uptake rates between these spores, although as expected (Korza et al., 2023), decoated spores had much higher methylamine permeability than intact spores (compare Supplementary Figures S3A,B).

### YetF and 2Duf homologs in *Firmicute,* and a conserved YetF fold

Given the large effects of *yetF* and *ydfS* deletions on *B. subtilis* spore properties, an obvious question concerns the distribution of these genes in *Firmicutes*, both spore forming and asporogenous species. To answer this question, a broad Blast search using the *B. subtilis* YetF sequence as a query identified 173 YetF homologs in 60 completed *Bacilli* genomes, with 1–8 genes per species (Supplementary Table S2 and Supplementary Figure S5). In addition, a similar search carried out on 58 annotated *Clostridia* genomes has identified 134 YetF homologs with 1–6 genes per species (Supplementary Table S3 and Supplementary Figure S6). As expected, all these homologs were identified as Duf421 domain-containing proteins. Importantly, the coding genes for YetF, YdfS and three other homologs (YkjA, YrbG, and YdfR) in *B. subtilis* exhibit expression almost exclusively during sporulation, and this expression parallels that of genes transcribed by the forespore-specific RNA polymerase with σ^G^ or σ^F^ (see transcription browser in SubtiWiki). This pattern of gene expression is consistent with the reported transcription of *yetF* by σ^F^ and *ydfS* and *ydfR* by σ^G^ (Arrieta-Ortiz et al., 2015). The *ykjA* and *yrbG* genes are also likely σ^F^ and σ^G^ dependent, respectively, as indicated by the presence of good matches to these respective promoter sequences in the genes’ upstream sequences (Supplementary Figure S4).

**Figure 9:**
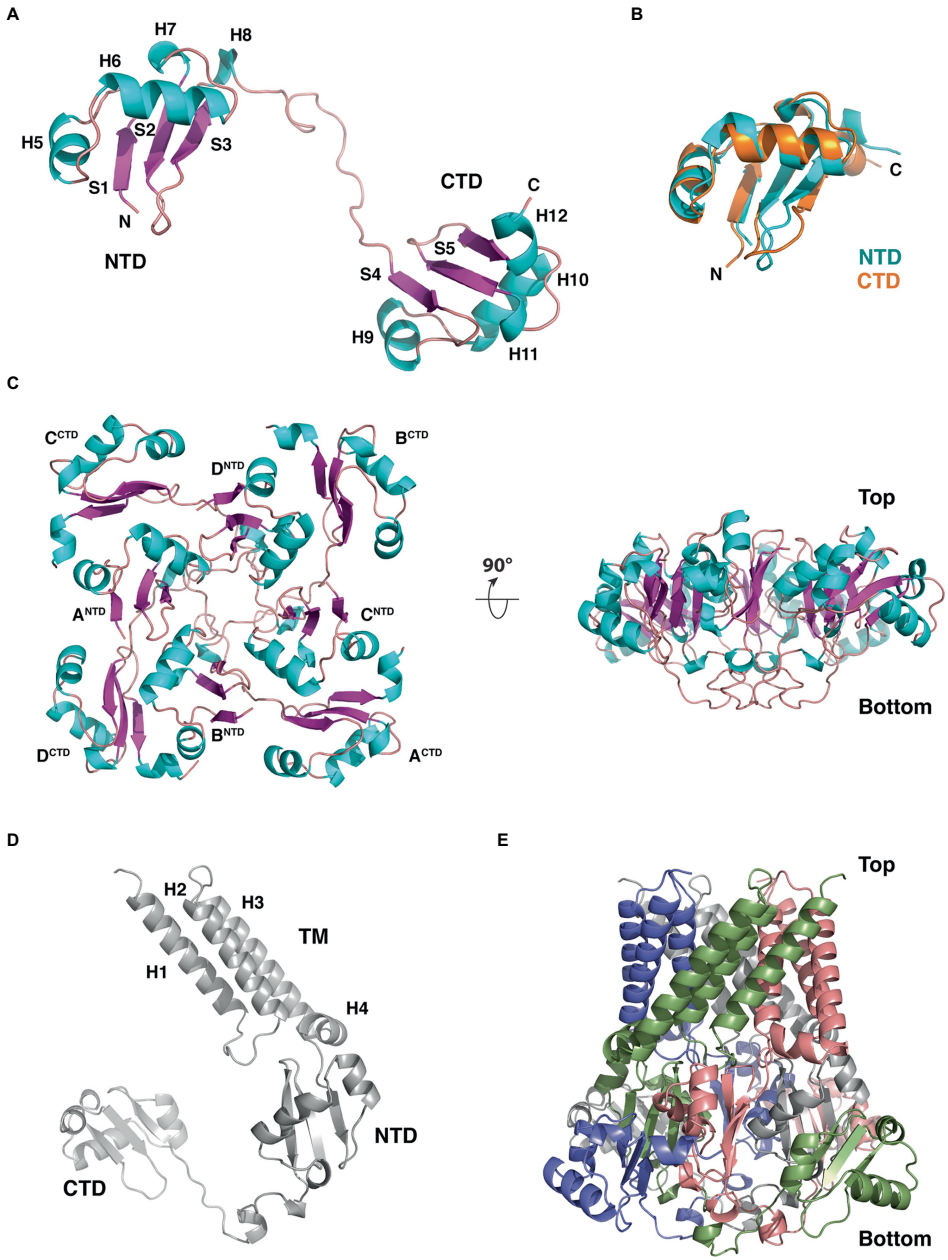
Structure, domain organization and oligomerization state of *B. subtilis* YetF. **(A)** Ribbon diagram of the YetF monomer lacking the N-terminal transmembrane helices (PDB ID: 3C6F), with the secondary-structure elements labeled. Helices and β strands are shown in blue and purple, respectively. **(B)** Superimposition of the NTD (blue) and CTD (orange) subdomains of YetF. **(C)** Top and side views of the YetF tetramer observed in the crystal structure. **(D)** Computed full-length YetF monomer structure by AlphaFold (Jumper et al., 2021) including the N-terminal TM helices. **(E)** Tetrameric assembly of the full-length YetF, shown in an orientation similar to that in (**C**, right).

The crystal structure of *B. subtilis* YetF lacking the N-terminal hydrophobic helices (residues 94–231) was recently determined by the New York SGX Research Center for Structural Genomics (NYSGXRC) at a resolution of 2.5-Å (PDB ID: 3C6F; Figure 9). YetF adopts a dumbbell-shaped fold consisting of two globular N- and C-terminal subdomains (NTD and CTD) connected by a 20-residue flexible linker (Figure 9). Each subdomain contains a central three-stranded antiparallel β-sheet flanked by four helices (Figure 9). Despite their low sequence identity (17.9%), the NTD and CTD domains share essentially identical secondary structure topology and connectivity and superimpose very well with a 1.27 Å root-mean-square deviation over 56 aligned C_α_ positions (Figure 9B). Interestingly, there are four copies of YetF in the asymmetric unit forming a homo-tetramer (Figure 9C). Viewed down the four-fold axis, the YetF tetramer has the overall architecture of a pinwheel, consisting of a tightly packed core formed from the NTDs of each monomer and protrusions formed by the respective CTDs; each NTD interacts with the CTD of the monomer from the opposite side (Figure 9C). This tetrameric assembly of YetF encloses a vase-shaped central pore connecting the NTDs on one end and the linker of the two subdomains on the other end. The association of the four YetF monomers buries a total of ~12,680 Å^2^ of solvent-accessible surface, ~42% of the monomer surface, suggesting that YetF likely functions as a tetramer.

The extreme N-terminal region of YetF is conserved in all Duf421-containing proteins and is predicted to contain multiple TM helices spanning the spore IM. We thus used AlphaFold (Jumper et al., 2021) to compute the structural model of the full-length YetF (Figure 9D). As expected, the predicted transmembrane motif is highlighted with three long hydrophobic helices that project away from two other subdomains of YetF (Figures 1, 9D). We note that the predicted NTD and CTD models of YetF are highly similar to the crystal structure. We next constructed the full-length YetF tetramer using the crystal structure as the template. As a result, the transmembrane helices together with the rest of the protein form a highly unusual basket-like arrangement with a central pore, likely anchored to the spore IM (Figure 9E). A hierarchical search against the Protein Data Bank using Dali (Holm, 2020) failed to reveal any other proteins with a topology significantly similar to that of YetF, suggesting that YetF has a novel fold. Importantly, the overall fold of the four other YetF homologs in *B. subtilis* predicted using AlphaFold (Jumper et al., 2021) closely resembles the structure observed for YetF (data not shown), suggesting that the core fold of YetF likely represents a common fold for all Duf421 domain-containing proteins.

**Figure 10:**
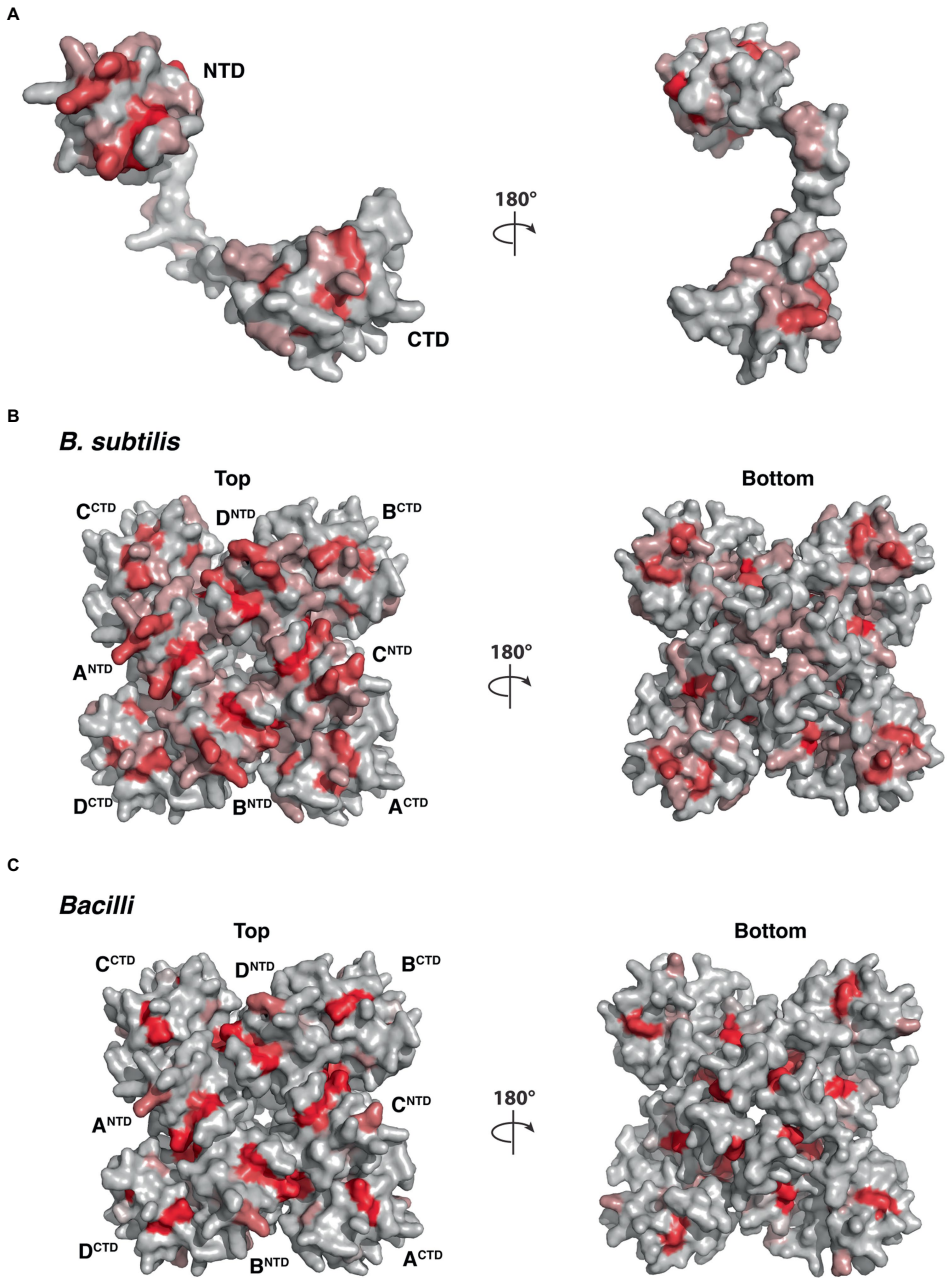
Molecular surface of YetF (PDB ID: 3C6F) colored according to homologous amino acid sequence conservation. The colors vary from dark red (highly conserved residues) to light gray (least conserved). **(A,B)** Surface representation of YetF as a monomer **(A)** and a tetramer **(B)**, showing conservation among YetF, YdfS and three other YetF homologs in *B. subtilis* (see also Figure 1). **(C)** Surface representation of a tetrameric YetF, showing conservation among best matched YetF homologs identified in other *Bacilli* as shown in Supplementary Figure S5.

To illustrate the homologous sequence conservation in the structural context, we mapped the conservation of the five YetF homologs in *B. subtilis* onto the surface of the YetF structure. As shown in Figure 10, the NTD and CTD subdomains of YetF share higher conservation than the linker region. Consistent with this observation, the residues on the top face of the tetrameric “basket” lined with residues from the NTD of YetF are more conserved than the bottom face with the linker region (Figure 10B). Moreover, the interfaces between the NTD and its neighboring CTD are also more conserved, suggesting that forming a tetramer may play important roles in mediating specific functions of YetF-like proteins. Importantly, the similar conservation trends are observed in the representative YetF homologs identified from other *Bacilli* and *Clostridia* species (Figure 10C and Supplementary Figure S7).

While searching for YetF homologs in other *Bacilli* and *Clostridia*, we found that the hits can be categorized into two groups, based on the molecular weights - close to YetF or to 2Duf (Table 2). The YetF homologs contain only a Duf421-like domain, while 2Duf homologs have both Duf421- and Duf1657-like domains. Among other *Bacilli* species examined that have at least one match to *yetF*, these spore formers have an average of ~3 YetF homologs, while genomes of almost all asporogenous *Bacilli* species with a match to YetF only have one homolog (Supplementary Table S2). On the other hand, the average number of YetF homologs in well documented spore-forming *Clostridia* species with at least one match to *B. subtilis* YetF was 1.6, lower than in *Bacilli* (Supplementary Table S3). Notably, the genomes of some *Clostridia* species thought to be asporogenous and yet having 1–6 YetF homologs also had *gpr*, *spoVFA* and *spoVFB* genes (data not shown), part of the sporulation specific (*spo*) group of genes associated with spore formation (Galperin et al., 2022), suggesting these species could be spore formers (Table 2 and Supplementary Table S3). Three reportedly asporogenous *Clostridia* species that did not contain the three *spo* genes noted above also have *yetF* genes, but only one (Table 2 and Supplementary Table S3).

While the *B. subtilis* wt genome does not contain a 2Duf homolog, ~50% of the spore-forming *Bacilli* surveyed possesses one or two copies of 2Duf homologs, but with no *2duf* gene in the asporogenous *Bacilli* (Supplementary Table S2). On the other hand, six (~18%) of well documented spore-forming *Clostridia* have a single 2Duf homolog while having at least one YetF homolog, but none of the likely spore formers or asporogenous species has any (Table 2 and Supplementary Table S3). Our analyses support the notion that both YetF and 2Duf homologs are widespread in *Bacilli* and *Clostridia* species and likely play a major role in spore wet heat resistance and germination in spores of all species.

### Where did the *2duf* gene come from?

**Figure 11:**
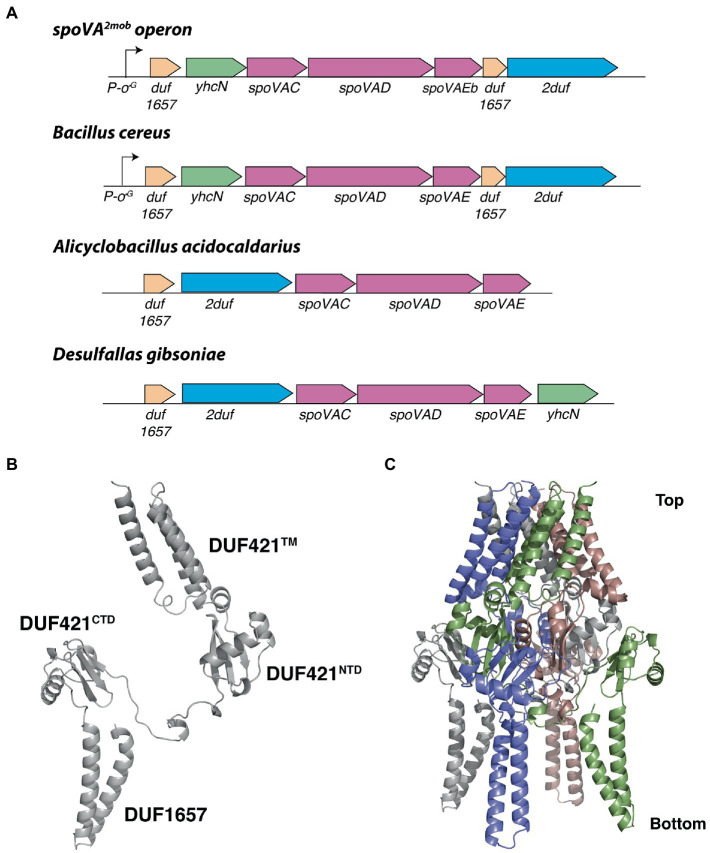
Local gene organization of representative 2*duf* homologs and computed 2Duf structures. **(A)** Overview of the *spoVA*^2mob^ and representative *2duf* homologs identified in *Bacilli* and *Clostridia*, shown in their genetic context. Both the *spoVA*^2mob^ and the *2duf* homolog from *B. cereus* have a predicted σ^G^ promoter binding site upstream of the first gene. **(B)** Computed 2Duf monomer structure by AlphaFold (Jumper et al., 2021) including the Duf421-like domain (TM, NTD, and CTD) and the Duf1657 domain, shown in an orientation similar to that in Figure 9D. **(C)** Tetrameric assembly of 2Duf, shown in an orientation similar to that in **(B)**.

Having learned that the *2duf* gene is present in many *Bacilli* species, an obvious question is whether one might be the source of the *2duf* gene that was picked up by a transposon to give the extremely high wet heat-resistant spores found in a food processing plant (Berendsen et al., 2016a)? To attempt to answer this question, the percent sequence identities between the 2Duf encoded in *spoVA*^2mob^, and those encoded in various *Bacilli* species were determined (Supplementary Table S4). The highest percent identities with the 2Duf homologs were encoded by the closely related *Bacilli* species, *B. cereus, B. thuringiensis*, and *B. anthracis*. In addition to *2duf*, the last gene in the *spoVA*^2mob^ operon, this operon also carries three *spoVA* genes, *spoVAC*, *spoVAD*, and *spoVAEb* (Figure 11) which are sufficient to form a channel in spores’ IM essential for uptake of CaDPA in sporulation and its release in germination (Setlow et al., 2017; Luo et al., 2021). Notably, *B. cereus* has two *spoVA* operons, one with classic seven *spoVA* genes, and a three-gene operon like that in *spoVA*^2mob^ (Paredes-Sabja et al., 2011). Examination of the *B. cereus* genome around the three-gene *spoVA* operon found that *2duf* is downstream of the three-gene *spoVA* operon, separated from it by a gene encoding a Duf1657 present at this position in *spoVA*^2mob^, and the *spoVA* operon is preceded first by a *yhcN* gene and then another *duf1657* gene, also in these positions in *spoVA*^2mob^ (Figure 11). The *spoVA*^2mob^ operon is preceded and presumably transcribed from a σ^G^ promoter just before the first *duf1657* gene (Berendsen et al., 2016a), and the *duf1657* gene upstream of the *B. cereus* three-gene *spoVA* operon is also preceded by a similar likely σ^G^ promoter sequence (Figure 11) An identical genomic arrangement around *2duf* was also seen in the genomes of *B. anthracis* and *B. thuringiensis*, very close *B. cereus* relatives (Supplementary Table S4 and Figure 11). However, other species, including multiple *Clostridia* species, had different arrangements of *2duf*, although always near the three *spoVA* genes, as well as other genes found in *spoVA*^2mob^ (Figure 11 and Supplementary Table S4). Perhaps Duf1657 and YhcN and even the three SpoVA proteins play some role in 2Duf function in spores, although wt *B. subtilis’* genome has no additional three-gene *spoVA* operon, and y*etF* homologs are not adjacent to the seven-gene *spoVA* operon or to *yhcN* and *duf1657* genes (data not shown). Notably, the Duf421 domain in 2Duf has a predicted structure similar to that of YetF and the Duf1657 domain forms two long helices (Figure 11B). Using the YetF tetramer as the template, the assembled 2Duf tetrameric structure shares an overall arrangement very similar to that of YetF, including the TM helices above the basket presumably anchored to spores’ IM, and the Duf1657 helices adding a ‘stand’ for the basket-like structure (Figure 11C).

### Presence and relative levels of YetF homologs in growing cell and spore membranes

There are many publications reporting mass spectrometry analyses of the proteome of *B. subtilis*, *B. cereus* and *Clostridiodes difficile* vegetative cells and spores, including IM proteins (Zheng et al., 2016; Swarge et al., 2018; Abhyankar et al., 2019; Swarge et al., 2020; Gao et al., 2021, 2022). Such work identified all five YetF homologs in the *B. subtilis* spore IM (Zheng et al., 2016). The sum of spectral counts over three replicates to estimate the relative abundance of these five proteins in this sample out of the 1,325 *B. subtilis* IM proteins identified and ranked from most (1) to least (1325) abundant showed the order of ranking to be: YetF (113), YrbG (212), YkjA (562), YdfS (712), and YdfR (992). This ranking suggests that YetF is more abundant in spores than YdfS, consistent with the much larger effects of the *yetF* deletion than deletion of *ydfS* on spore properties seen in the current work. In addition, this same work did not find any of these five proteins in vegetative *B. subtilis* cells, which is consistent with their forespore-specific transcription noted above. Presumably these spore proteins are degraded in spore outgrowth following germination. The *B. cereus* homolog with the best match to *B. subtilis* YetF (UniProt ID Q813Q8), as well as a 2Duf homolog (UniProt ID Q812J9), were also present in the *B. cereus* spore IM proteome (iBAQ ranks 559 and 626, respectively out of 1,254), but not in vegetative cells or the vegetative cell membrane fraction (Gao et al., 2021). In addition, the one *C. difficile* spores’ 219 aa YetF homolog (UniProt ID Q187A0) was also identified in these spores’ IM proteome, with an approximate abundance at the median out of 1,021 proteins identified; this protein was also absent from the vegetative cell membrane fraction (Abhyankar et al., 2019).

## Discussion

This work shows that YetF and YdfS play major roles in *B. subtilis* spore properties, including their resistance and germination. Both proteins are found in spores’ IM, as are the other 3 *B. subtilis yetF* homologs, suggesting that it is either by altering IM properties that these proteins exert their effects, or by directly or indirectly altering properties of other IM proteins, such as GRs and/or the germinosome, since effects of these proteins on spore germination are on a step prior to CaDPA release. The work in this communication certainly indicates that YetF and YdfS alter spore IM properties as there are: (i) decreases in resistance to several DNA damaging chemicals consistent with concomitant increases in IM permeability when YetF is absent and smaller changes when YdfS is absent; (ii) decreases in wet heat resistance of at least one IM germination protein in YetF’s absence and less so when YdfS is absent; and (iii) decreases in GR-dependent germination, a process that requires multiple IM proteins, in the absence of YetF, and less so in the absence of YdfS. Given that homologs of this group of proteins are found in all spore-forming *Bacilli* as well as in spore forming *Clostridia*, it seems reasonable to expect that these proteins play major roles in the resistance and germination of spores of all *Firmicutes*, although further experimentation will be needed to prove this. It may be particularly interesting to examine the role of these proteins in *C. difficile* spore germination, as this species’ spore germination does not proceed through IM GRs (Setlow and Johnson, 2019).

A major question is how YetF/YdfS proteins as well as 2Duf, exert their effects. Since they appear to decrease IM permeability, one possibility is that this is what causes the increased spore wet heat and H_2_O_2_ resistance due to these proteins, as suggested recently (Kanaan et al., 2021; Korza et al., 2023). However, how these proteins alter IM permeability is not clear, and while methylamine permeability was not altered by the absence of YetF/YdfS, previous work showed that spore IM fluidity was increased when 2Duf was present (Korza et al., 2023). It is also possible that the effects on IM properties are how YetF/YdfS and 2Duf alter rates of GR-dependent spore germination. However, these proteins could also affect germination by direct effects on IM GRs or the IM complex of GRs plus the GerD protein termed the germinosome, formation of which increases spore germination rates >5-fold (Setlow et al., 2017).

Another possible role of YetF/YdfS and 2Duf is in the variability in wet heat resistance between individual spores in populations (Hornstra et al., 2009; Zhang et al., 2010), differences that can be extremely large. Indeed, stochastic variation of these proteins’ levels in individual spores in populations would be expected, as seen previously examining variation in *B. subtilis* spores’ levels of various GRs (Griffiths et al., 2011). There is also significant heterogeneity in *B. subtilis* germination kinetics between individuals in a population (Setlow et al., 2017), likely due to stochastic differences in levels of GRs, although variation in levels of YetF/YdfS could also contribute. In addition, an increase in YetF levels could explain the facile evolution of spores’ wet heat resistance seen recently with spores of *B. weihenstephanensis* (Kim et al., 2021), since only appropriate promoter mutations in *yetF* might be needed. Again, it will be important to determine: (i) the natural variation in levels of YetF homologs between individual spores in populations; (ii) whether such differences correlate with differences in rates of spore killing by wet heat or spore germination; and (iii) whether the easily evolved *B. weihenstephanensis* spores with high wet heat resistance have increased levels of YetF.

The large variation in numbers of YetF homologs in various *Bacilli* and *Clostridia* species as well as the presence of one or rarely two 2Duf homologs, all almost certainly in spores, also raises questions about how these various homologs together modulate spore’s rates of germination and levels of resistance, perhaps most importantly its wet heat resistance. Answering this question will require not only knowing how many of these various genes are in a spore former, but also the levels of the various proteins, something yet unknown. In addition, while it seems likely that YetF alone can modulate spore wet heat resistance, it is not yet clear that 2Duf alone can do this, as this gene is invariably surrounded by an identical group of other genes, whether in *spoVA*^2mob^ on a transposon or in genomes of many other species. Perhaps one or more of these acolytes is also involved in 2Duf function.

Finally, identification of the role YetF and its homologs play in spore resistance has certainly changed the way spores’ resistance to a variety of agents, including wet heat, H_2_O_2_ and other agents that must cross the IM must be viewed. This work has also generated a myriad of questions on how these proteins exert their effects either as a group or individuals. A sample of these questions include the following: (1) do these proteins affect germinosome assembly in dormant spores; (2) where in the IM are these proteins, are they throughout the IM or perhaps clustered with either the germinosome or the SpoVA channel for CaDPA or both; (3) what is the variation in the levels of these proteins in individual spores in population and does this correlate with these individual spores’ resistance and/or germination properties, and might changes in promoters of these genes give rise to strains making spores with more or less heat resistance than spores of the parental strain; (4) do the different YetF homologs play synergistic roles in spore properties or independent roles, and what are the effects of deleting all five homologs on spore resistance and germination properties; (5) what is the fate of these spore proteins in germination and outgrowth – are they rapidly degraded as are spores’ DNA protective proteins (Setlow, 1988), and if the latter, what enzyme is catalyzing the degradation; (6) do different YetF homologs have different roles in spore resistance; (7) what specific role do these proteins have in spore germination; and (8) what role if any do these proteins play in non-spore formers, as homologs of *yetF*/*ydfS* are found not only in spore forming *Firmicutes*, but also in asporogenous *Bacilli* species and perhaps asporogenous *Clostridia* species, although *yetF* homologs are not members of the stress response σ^B^ regulon in *B. subtilis* (Yeak et al., 2023). Given the ubiquity of these proteins in non-spore formers, it seems likely they may also do something important in growing or stationary phase cells. Clearly, many new fundamental questions have been raised by the finding of the large effects of YetF and YdfS on *B. subtilis* spore properties. Consequently, the discovery of this group of proteins in spores can provide work for molecular microbiologists for many years yet. Hopefully the corresponding authors will have many years left to do this!

## Data availability statement

The original contributions presented in the study are included in the article/Supplementary material, further inquiries can be directed to the corresponding authors.

## Author contributions

PS and BH conceived the project, administered, and supervised the research. PS wrote the first draft of the manuscript. BH wrote the material on YetF and 2Duf structure and sequence conservation. GKo supervised and carried out the research. BY carried out research and was the first to see the effects of the *yetF* deletion on spore wet heat resistance. JW, HS, and JK carried out research on spore killing, resistance, and germination. FN carried out phospholipid analyses, a line of investigation suggested by SB. YL and BH made and validated constructs expressing *yetF* at various loci. BH carried out the bioinformatic analyses with PS and with YL prepared the tables and figures with these data. GKr and PS analyzed published proteomic data from spores and vegetative cells and their membranes. Y-qL supervised the obtaining of individual spore germination data by MA, and prepared the table and figures from these experiments. All authors have read and edited the final version of the manuscript.

## Conflict of interest

The authors declare that the research was conducted in the absence of any commercial or financial relationships that could be construed as a potential conflict of interest.

## Publisher’s note

All claims expressed in this article are solely those of the authors and do not necessarily represent those of their affiliated organizations, or those of the publisher, the editors and the reviewers. Any product that may be evaluated in this article, or claim that may be made by its manufacturer, is not guaranteed or endorsed by the publisher.

## Footnotes

^1^ https://bgsc.org/_catalogs/Catpart4.pdf

^2^ http://subtiwiki.uni-goettingen.de/
