# Supplementary material for: Identification and characterization of new proteins crucial for bacterial spore resistance and germination
Source: Front Microbiol. 2023 Apr 11;14:1161604. doi: 10.3389/fmicb.2023.1161604 (PMC10126465; doi:10.3389/fmicb.2023.1161604)
Supplement: Supplementary file 1 [file Data_Sheet_1.pdf]

Supplementary Information

Identification and Characterization of New Proteins Crucial for Bacterial Spore Resistance and Germination

Benjamin Yu[1], Julia Kanaan[1], Hannah Shames[1], James Wicander[1], Makunda Aryal[2], Yunfeng Li[1], George Korza[1], Stanley Brul[3], Gertjan Kramer[4], Yong-qing, Li[2], Frank C. Nichols[5], Bing Hao[1] and Peter Setlow*[1]

[1]Department of Molecular Biology and Biophysics, and [5]Division of Periodontology, UConn Health, Farmington, CT 06030-3305, USA; [2]Department of Physics, East Carolina University, Greenville, NC 27858-4353, USA; [4]Molecular Biology & Microbial Food Safety and Laboratory for Mass Spectrometry of Biomolecules, Swammerdam Institute for Life Science, University of Amsterdam, 1098XH Amsterdam, The Netherlands

Supplementary Information List

1. Supplementary Table S1
2. Supplementary Table S2
3. Supplementary Table S3
4. Supplementary Table S4
5. Supplementary Figure S1
6. Supplementary Figure S2
7. Supplementary Figure S3
8. Supplementary Figure S4
9. Supplementary Figure S5
10. Supplementary Figure S6
11. Supplementary Figure S7

Supplementary Table S1 Average parameters of the germination of multiple individual wt and *yetF* spores

Spores	PS832		PS4488	
	Valine	AGFK	Valine	AGFK
T_{lag}	20.3 ± 8.9	36.5 ± 16.6	44.6 ± 23.1	49.0 ± 20.5
$T_{release}$	24.0 ± 9.4	40.3 ± 16.9	48.2 ± 23.0	53.8 ± 20.9
T_{lys}	34.7 ± 9.2	52.0 ± 16.7	63.6 ± 17.6	74.5 ± 11.5
$\Delta T_{release}$	3.7 ± 1.4	3.8 ± 1.1	3.6 ± 1.2	4.8 ± 1.6
ΔT_{lys}	10.7 ± 2.8	11.7 ± 4.7	15.4 ± 8.0	20.7 ± 11.5
I_{lag}	0.88 ± 0.07	0.83 ± 0.06	0.77 ± 0.07	0.76 ± 0.09
$I_{release}$	0.21 ± 0.05	0.29 ± 0.07	0.29 ± 0.07	0.23 ± 0.07
ger/num†	97/139	80/104	44/133	14/40

Spores were germinated and individual germination parameters were measured as described in Methods. †Number of spores that germinated/number of spores examined.

Supplementary Table S2 Homologs of *B. subtilis yetF* or *2Duf* in completed genomes of *Bacilli*.

Bacilli spore forming species	Number of homologs to		Asporogenous *Bacilli* species	Number of homologs to	
	yetF	*2duf*		*yetF*	*2duf*
Aeribacillus pallidus	1	1	*Aerococcaceae bacterium*	1	0
Alicyclobacillus acidocaldaruis	0	1	*Auricoccus indicus*	1	0
Amphibacillus xylanus	2	0	*Brochothrix thermosphacta*	1	0
Aneurinibacillus soli	0	1	*Carnobacterium maltaromaticum*	1	0
Anoxyacillus flavithermus	2	1	*Enterococcus faecalis*	1	0
Bacillus amyloliquefaciens	1	0	*Gemella haemolysans*	1	0
Bacillus anthracis	3	1	*Jeotgalibaca dankookensis*	2	0
Bacillus atrophaeus	1	0	*Lactobacillus acidophilus*	1	0
Bacillus cereus	5	1	*Lactobacillus plantarum*	1	0
Bacillus firmus	1	0	*Lactobacillus reuteri_*	1	0
Bacillus halodurans	8	2	*Lactobacillus rhamnosus*	1	0
Bacillus licheniformis	6	0	*Lactococcus lactis*	1	0
Bacillus megaterium	1	0	*Leuconostoc kimchii*	1	0
Bacillus pumilus	1	0	*Marinilactibacillus 15R*	1	0
Bacillus subtilis	5	0	*Melissococcus plutonius*	1	0
Bacillus thuringiensis	5	1	*Planococcus antarcticus*	1	0
Bacillus weihenstephanensis	1	0	*Salimicrobium jeotgali*	1	0
Brevibacillus brevis	4	1	*Salinicoccus halodurans*	2	0
Cohnella candidum	5	1	*Streptococcus mutans GS-5*	1	0
Fictibacillus phosphorivorans	6	0	*Streptococcus pneumoniae*	1	0
Geobacillus kaustophilus	0	1	*Vagococcus penaei*	1	0
Geobacillus stearothermophilus	1	0			
Halobacillus halophilus	6	1			
Kyrpidia tusciae	0	2			
Laceyella sacchari	1	1			
Lentibacillus amyloliquefaciens	3	1			
Lysinibacillus sphaericus	4	0			
Novibacillus thermophilus	3	0			
Oceanobacillus iheyensis	2	1			
Paenibacillus polymyxa	2	1			
Paenisporosarcina cavernae	3	0			
Parageobacillus thermoglucosidasius	1	1			
Paraliobacillus zengyii	8	0			
Solibacillus silvestris	4	0			
Sporolactobacillus terrae	5	0			
Sporosarcina psychrophila	3	0			
Terribacillus goriensis	4	0			
Thermobacillus composti	2	0			
Virgibacillus halodenitrificans	3	2			
**Average number in ones with ≥ 1**	**3.2**	**1**	**Average number per species**	**1.1**	**0**

Supplementary Table S3 Homologs of *B. subtilis yetF/2Duf* in *Clostridia* genomes.

Clostridia spore formers	Number of homologs to		Likely *Clostridia* spore formers (have *gpr, spoVFA, spoVFB)*	Number of homologs to	
	yetF	*2duf*		*yetF*	*2duf*
Alkaliphilus metalliredigens	3	1	*Acetohalobium arabaticum*	5	0
Ammonifex degensii	2	0	*Anoxybacter fermentans*	2	0
Caldanaerobacter subterraneus	1	0	*Caldicellulosiruptor saccharolyticus*	1	0
Candidatus Arthromitus sp. SFB-mouse	1	0	*Carboxydocella thermautotrophica*	2	0
Candidatus Desulforudis audaxviator	1	0	*Ethanoligenens harbinense*	4	0
Caproiciproducens sp. NJN-50	2	0	*Faecalibacterium prausnitzii*	1	0
Cellulosilyticum lentocellum	1	0	*Flavonifractor plautii*	1	0
*Clostridioides difficile_*	1	0	*Halobacteroides halobius*	6	0
Clostridium acetobutylicum	5	0	*Halocella sp. SP3-1*	5	0
Clostridium botulinum	2	0	*Halothermothrix orenii*	6	0
Clostridium perfringens	1	0	*Herbinix luporum*	2	0
Clostridium tetani	2	0	*Natranaerobius thermophilus*	2	0
Dehalobacterium formicoaceticum	1	1	*Pelotomaculum thermopropionicum*	2	0
Desulfallas gibsoniae	2	1	*Ruminococcus albus*	1	0
Desulfitobacterium hafniense	3	0	*Syntrophomonas wolfei*	3	0
Desulfofarcimen acetoxidans	3	0	*Syntrophothermus lipocalidus*	2	0
Desulfofundulus kuznetsovii	2	0	*Thermincola potens*	3	0
Desulfosporosinus orientis	2	1	**Average number in ones with ≥1**	**2.8**	**0**
Desulfotomaculum ruminis	3	1			
Hungateiclostridium thermocellum	1	0	*Asporogenous Clostridia species*		
Intestinimonas butyriciproducens	1	0	*Aminipila sp. JN-39*	1	0
Lachnoanaerobaculum umeaense	2	0	*Carboxydothermus hydrogenoformans*	1	0
Lachnoclostridium phytofermentans	1	0	*Dehalobacter sp. CF*	1	0
Mahella australiensis	1	0	*Thermosediminibacter oceani*	1	0
Moorella thermoacetica	1	0	**Average number per species**	**1**	**0**
Paeniclostridium sordellii	2	0			
Pseudoclostridium thermosuccinogenes	1	1			
Ruminiclostridium cellulolyticum	3	0			
Symbiobacterium thermophilum	2	0			
Syntrophobotulus glycolicus	1	0			
Tepidanaerobacter acetatoxydans	1	0			
Thermacetogenium phaeum	2	0			
Thermoanaerobacter italicus	1	0			
Thermoclostridium stercorarium	1	0			
**Average number in ones with ≥1**	**1.6**	**1**			

Supplementary Table S4 Sequence identity between 2Duf homologs in *Bacilli* and *Clostridia* and 2Duf encoded in *spoVA*^{2mob}.

Bacilli	NCBI ID	Identity to 2Duf (%)
**Group 1**		
Aeribacillus pallidus	WP_066246610.1	55.2
Aneurinibacillus soli	WP_096463052.1	50.9
Anoxyacillus flavithermus	WP_012575624.	55.1
Bacillus anthracis	WP_000018934	59.1
Bacillus cereus	WP_000018922.1	60.5
Bacillus halodurans	WP_010897735.1	30.5
Bacillus thuringiensis	WP_000018924.1	60.8
Brevibacillus brevis	WP_015892354.1	52.4
Cohnella candidum	WP_123042938.1	53.1
Geobacillus kaustophilus	WP_011230358	54.5
Halobacillus halophilus	WP_014642772.1	57.3
Lentibacillus amyloliquefaciens	WP_068440573.1	55.1
Oceanobacillus iheyensis	WP_011065783.1	50.9
Parageobacillus thermoglucosidasius	WP_003252282.1	55.6
Virgibacillus halodenitrificans	WP_019376107.1	59.6
**Group 2**		
Alicyclobacillus acidocaldaruis	WP_041694969	48.6
Kyrpidia tusciae	WP_013075904.1	38.1
Laceyella sacchari	WP_102991708.1	47.9
**Group 3**		
Paenibacillus polymyxa	WP_013370894.1	39.9
Clostridia		
Alkaliphilus metalliredigens	WP_012065254.1	42.0
Dehalobacterium formicoaceticum	WP_089611718.1	54.0
Desulfallas gibsoniae	WP_006522674.1	46.5
Desulfosporosinus orientis	WP_014185970.1	38.6
Desulfotomaculum ruminis	WP_013842559.1	44.8
Pseudoclostridium thermosuccinogenes	WP_103081847.1	39.9

Groups 1, 2 and 3 have the genomic arrangements around *2duf* in *B. cereus, A. acidocaldarius* and *D. gibsoniae*, respectively, in Figure 11A.

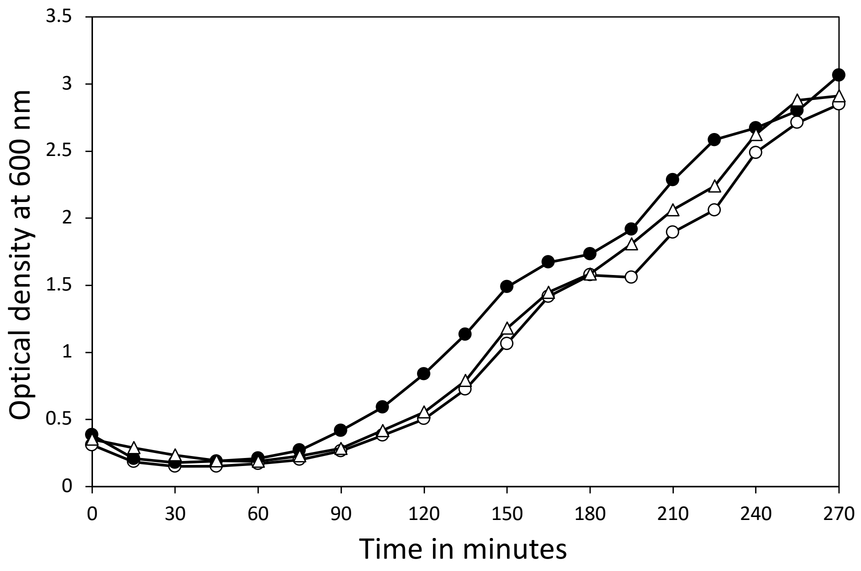

Supplementary Figure S1

Germination, outgrowth, and vegetative growth from dormant spores of various strains inoculated at ~10^8/ml in LB medium, incubated at 37°C and the culture's OD_{600} measured. The symbols used are: ○ – PS832; ● – PS4487; and △ – PS4488. A duplicate experiment found that all three strains again gave similar results.

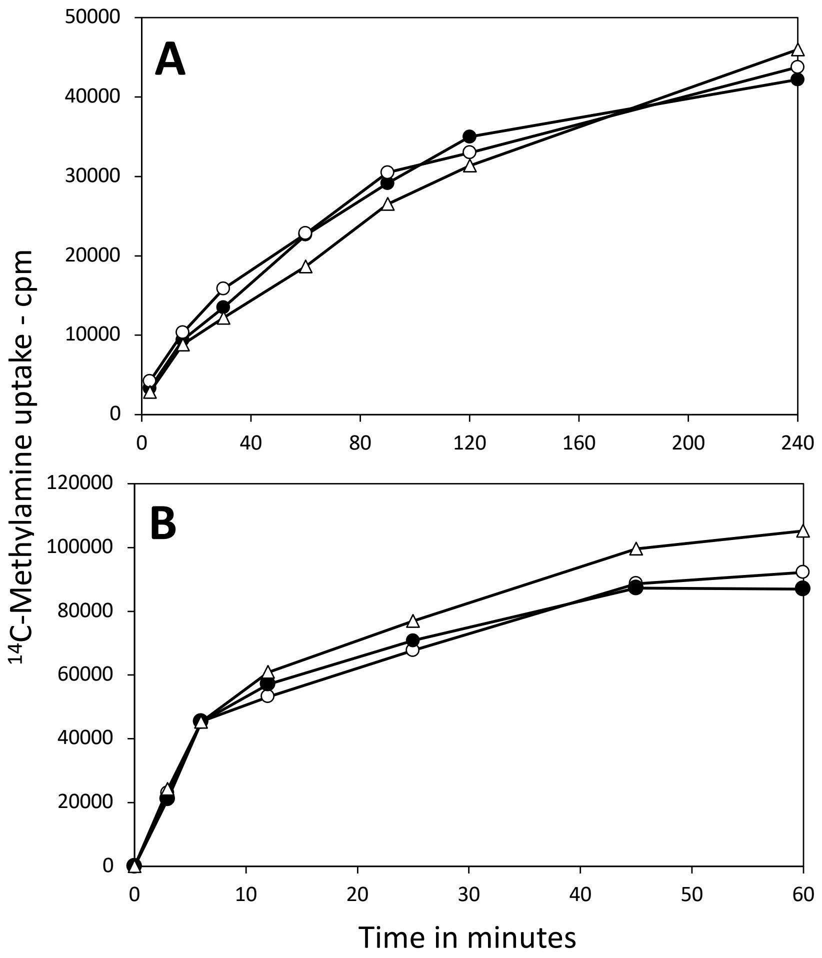

Supplementary Figure S2

^{14}C-Methylamine uptake by (**A**) intact and (**B**) decoated spores of PS832 (wt), PS4487 (*ydfS*) and PS4488 (*yetF*) spores. ^{14}C-Methylamine uptake by spores of various strains, either intact or decoated, was as described in Methods. Symbols used are: ○ – PS832; ● – PS4487; and △ – PS4488. These results were from single measurements in one experiment.

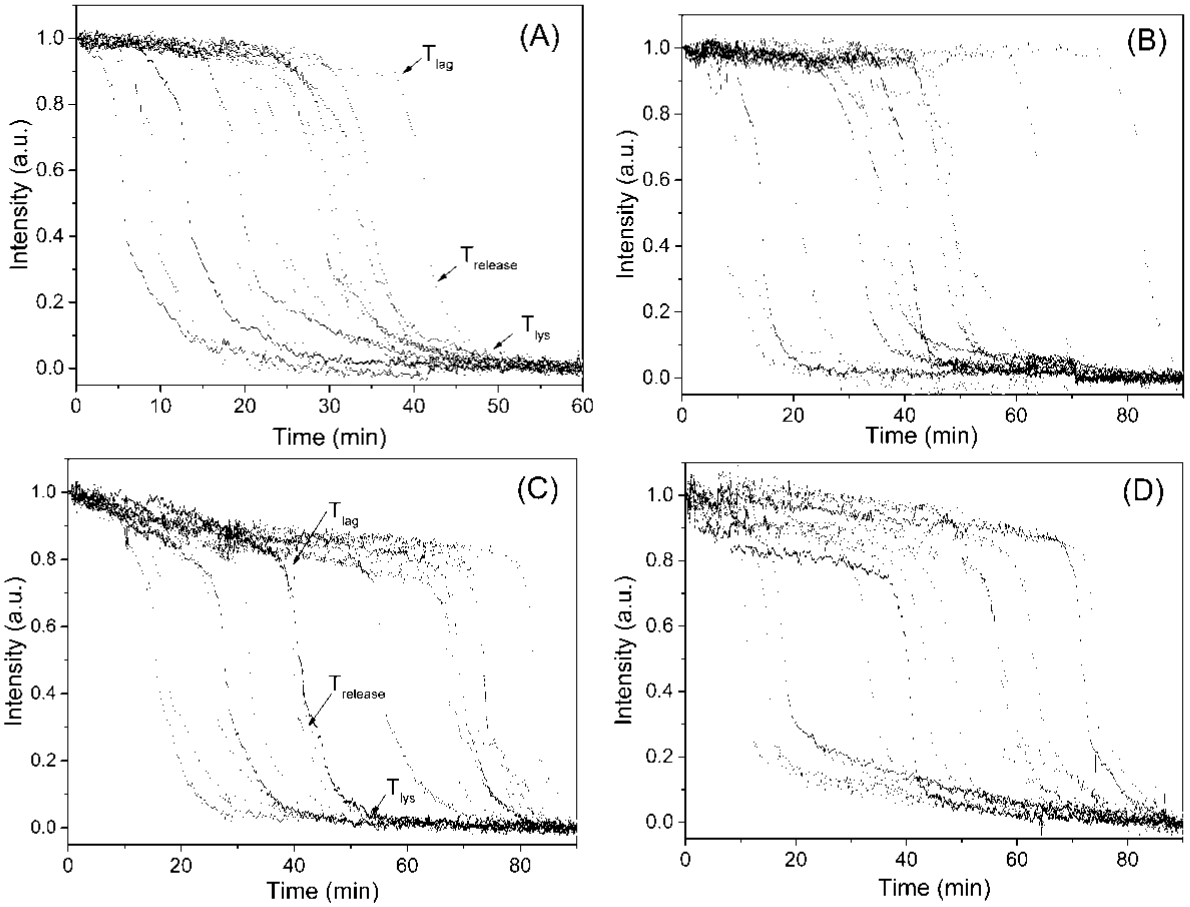

Supplementary Figure S3

Germination of multiple individual (**A,B**) PS832 (wt) and (**C,D**) PS4488 (*yetF*) spores germinating with (**A,C**) L-valine or (**B,D**) AGFK. Spores were heat-activated and germinated with L-valine or AGFK and the germination of 16 individual spores of each strain and with each germinant were followed as described in Methods. Arrows on one trace in panels (**A**) and (**C**) denote the times of T_{lag}, $T_{release}$ and T_{lys} and I_{lag} and I_{lys} were measured at T_{lag} and T_{lys}, respectively.

sspA

-35 **σ^{G}-10**

CAATTTTCAGCTCCTGTATACAATTACCAAAGTTTTTCT**GAATG**AAGCCATGTGTTTTGACA**CATTCTA**TACTC

RBS **TSC**

AC**AAGGAGGTG**AGACAC**ATG**GCTAACA

ykjA

-35 **σ^{F}** **-10**

TGTTCAGGCGATATGCACGTTCGCCTAGACACTGAT**GCATA**GAGGGCCTGCCACG**GG**G**CACAATA**GGCTC

RBS **TSC**

AATATGACCAGCGG**ATGGAGGTA**AAGATCG**GTG**TTATGGA

yrbG

-35 **σ^{G}-10**

GACGTAAAAGCTTTGT**GAAAT**GCAATCGGTTTTTCTTA**CATAAAT**TGATCATTTTGTTGCAACCTATAAT

RBS **TSC**

GAG**TTGGAGGTG**CATA**ATG**GAAGAGC

Supplementary Figure S4

Likely promoter sequences for the *ykjA* and *yrbG* genes and comparison with the very strong σ^{G}-dependent promoter sequence of *sspA*. The *sspA*, *ykjA* and *yrbG* upstream sequences include: -35 and -10 promoter sequences; a GG sequence just upstream of the putative -10 promoter sequence for y*kjA* indicative of a σ^{F} promoter; the ribosome binding sites (RBS) that were identified based on the optimal *B. subtilis* RBS sequence (AAGGAGGTG) and the translation start codon (TSC). The *sspA* promoter sequences were experimentally determined, and the promoter sequences for *ykjA* and *yrbG* were chosen to give the best matches to consensus σ^{F} and σ^{G} promoter sequences, respectively (Wang et al., 2006).

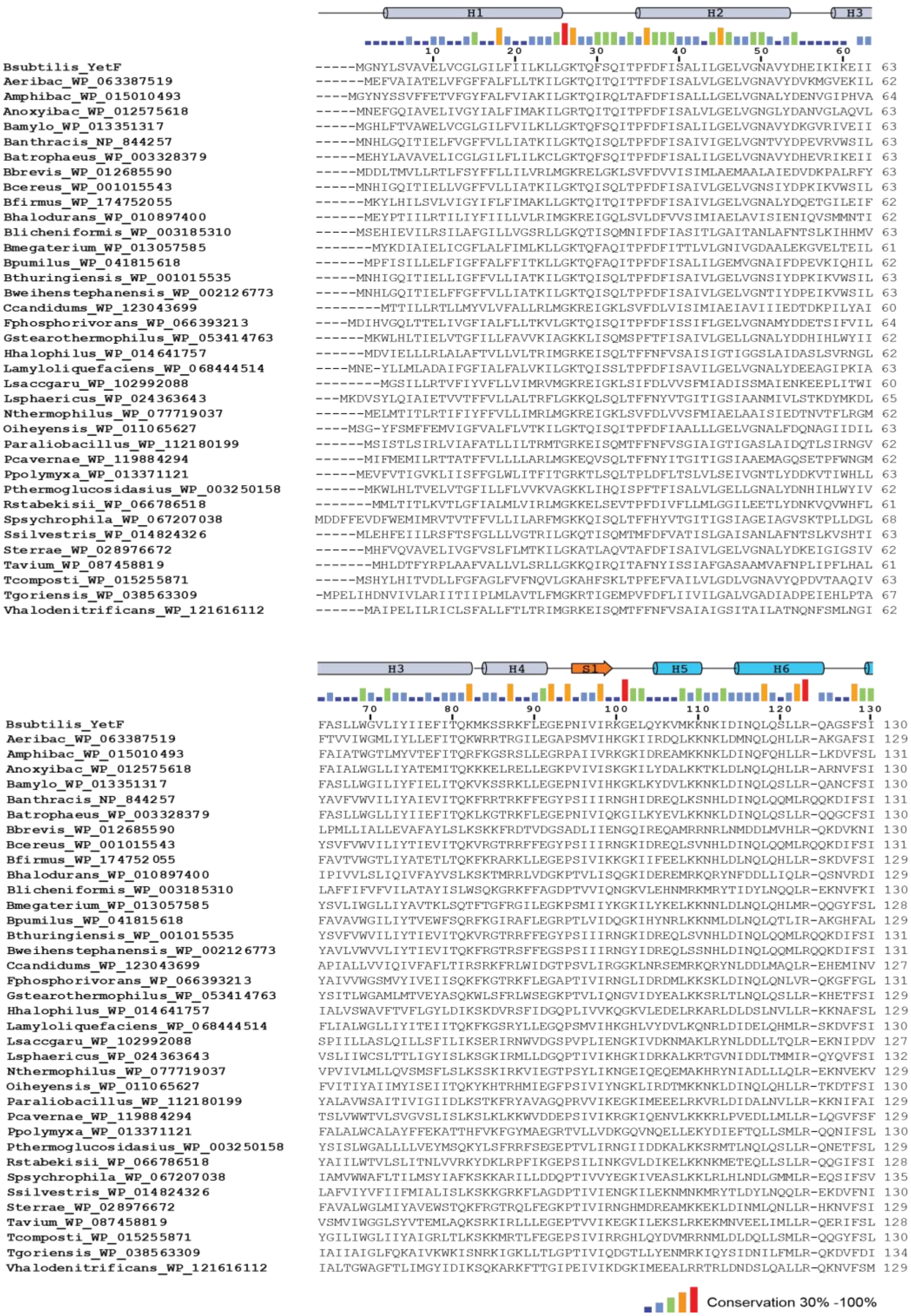
H1
H2
H3
10 20 30 40 50 60
Bsubtilis_YetF -----MGNYLSVAVELVCGLGILFIILKLLGKTQFSQITPFDFISALILGELVGNAVYDHEIKIKEII 63
Aeribac_WP_063387519 ------MEFVAIATELVFGFFALFLLTKILGKTQITQITTFDFISALVLGELVGNAVYDVKMGVEKIL 62
Amphibac_WP_015010493 ----MGYNYSSVFFETVFGYFALFVIAKILGKTQIRQLTAFDFISALLLGELVGNALYDENVGIPHVA 64
Anoxyibac_WP_012575618 -----MNEFGQIAVELIVGYIALFIMAKILGRTQITQITPFDFISALVLGELVGNGLYDANVGLAQVL 63
Bamylo_WP_013351317 -----MGHLFTVAWELVCGLGILFVILKLLGKTQFSQITPFDFISALILGELVGNAVYDKGVRIVEII 63
Banthracis_NP_844257 -----MNHLGQITIELFVGFFVLLIATKILGKTQISQLTPFDFISAIVIGELVGNTVYDPEVRVWSIL 63
Batrophaeus_WP_003328379 -----MEHYLAVAVELICGLGILFLILKCLGKTQFSQITPFDFISALILGELVGNAVYDHEVRIKEII 63
Bbrevis_WP_012685590 -----MDDLTMVLLRTLFSYFFLLILVRLMGKRELGKLSVFDVVISIMLAEMAALAIEDVDKPALRFY 63
Bcereus_WP_001015543 -----MNHIGQITIELLVGFFVLLIATKILGKTQISQLTPFDFISAIVLGELVGNSIYDPKIKVWSIL 63
Bfirmus_WP_174752055 ------MKYLHILSVLVIGYIFLFIMAKLLGKTQITQITPFDFISAIVLGELVGNALYDQETGILEIF 62
Bhalodurans_WP_010897400 ------MEYPTIILRTILIYFIILLVLRIMGKREIGQLSVLDFVVSIMIAELAVISIENIQVSMMNTI 62
Blicheniformis_WP_003185310 -----MSEHIEVILRSILAFGILLVGSRLLGKQTISQMNIFDFIASITLGAITANLAFNTSLKIHHMV 63
Bmegaterium_WP_013057585 -------MYKDIAIELICGFLALFIMLKLLGKTQFAQITPFDFITTLVLGNIVGDAALEKGVELTEIL 61
Bpumilus_WP_041815618 ------MPFISILLELFIGFFALFFITKLLGKTQFAQITPFDFISALILGEMVGNAIFDPEVKIQHIL 62
Bthuringiensis_WP_001015535 -----MNHIGQITIELLIGFFVLLIATKILGKTQISQLTPFDFISAIVLGELVGNSIYDPKIKVWSIL 63
Bweihenstephanensis_WP_002126773 -----MNHLGQITIELFFGFFVLLIATKILGKTQISQLTPFDFISAIVLGELVGNTIYDPEIKVWSIL 63
Ccandidums_WP_123043699 --------MTTILLRTLLMYVLVFALLRLMGKREIGKLSVFDLVISIMIAEIAVIIIEDTDKPILYAI 60
Fphosphorivorans_WP_066393213 ----MDIHVGQLTTELIVGFIALFLLTKVLGKTQISQITPFDFISSIFLGELVGNAMYDDETSIFVIL 64
Gstearothermophilus_WP_053414763 ------MKWLHLTIELVTGFILLFAVVKIAGKKLISQMSPFTFISAIVLGELLGNALYDDHIHLWYII 62
Hhalophilus_WP_014641757 ------MDVIELLLRLALAFTVLLVLTRIMGRKEISQLTFFNFVSAISIGTIGGSLAIDASLSVRNGL 62
Lamyloliquefaciens_WP_068444514 ----MNE-YLLMLADAIFGFIALFALVKILGKTQISSLTPFDFISAVILGELVGNALYDEEAGIPKIA 63
Lsaccgaru_WP_102992088 --------MGSILLRTVFIYVFLLVIMRVMGKREIGKLSIFDLVVSFMIADISSMAIENKEEPLITWI 60
Lsphaericus_WP_024363643 ---MKDVSYLQIAIETVVTFFVLLALTRFLGKKQLSQLTFFNYVTGITIGSIAANMIVLSTKDYMKDL 65
Nthermophilus_WP_077719037 ------MELMTITLRTIFIYFFVLLIMRLMGKREIGKLSVFDLVVSFMIAELAAISIEDTNVTFLRGM 62
Oiheyensis_WP_011065627 ----MSG-YFSMFFEMVIGFVALFLVTKILGKTQISQITPFDFIAALLLGELVGNALFDQNAGIIDIL 63
Paraliobacillus_WP_112180199 ------MSISTLSIRLVIAFATLLILTRMTGRKEISQMTFFNFVSGIAIGTIGASLAIDQTLSIRNGV 62
Pcavernae_WP_119884294 ------MIFMEMILRTTATFFVLLLLARLMGKEQVSQLTFFNYITGITIGSIAAEMAGQSETPFWNGM 62
Ppolymyxa_WP_013371121 -----MEVFVTIGVKLIISFFGLWLITFITGRKTLSQLTPLDFLTSLVLSEIVGNTLYDDKVTIWHLL 63
Pthermoglucosidasius_WP_003250158 ------MKWLHLTVELVTGFILLFLVVKVAGKKLIHQISPFTFISALVLGELLGNALYDNHIHLWYIV 62
Rstabekisii_WP_066786518 -------MMLTITLKVTLGFIALMLVIRLMGKKELSEVTPFDIVFLLMLGGILEETLYDNKVQVWHFL 61
Spsychrophila_WP_067207038 MDDFFEVDFWEMIMRVTVTFFVLLILARFMGKKQISQLTFFHYVTGITIGSIAGEIAGVSKTPLLDGL 68
Ssilvestris_WP_014824326 -----MLEHFEIILRSFTSFGLLLVGTRILGKQTISQMTMFDFVATISLGAISANLAFNTSLKVSHTI 63
Sterrae_WP_028976672 ------MHFVQVAVELIVGFVSLFLMTKILGKATLAQVTAFDFISAIVLGELVGNALYDKEIGIGSIV 62
Tavium_WP_087458819 -------MHLDTFYRPLAAFVALLVLSRLLGKKQIRQITAFNYISSIAFGASAAMVAFNPLIPFLHAL 61
Tcomposti_WP_015255871 -----MSHYLHITVDLLFGFAGLFVFNQVLGKAHFSKLTPFEFVAILVLGDLVGNAVYQPDVTAAQIV 63
Tgoriensis_WP_038563309 -MPELIHDNVIVLARIITIIPLMLAVTLFMGKRTIGEMPVFDFLIIVILGALVGADIADPEIEHLPTA 67
Vhalodenitrificans_WP_121616112 ------MAIPELILRICLSFALLFTLTRIMGRKEISQMTFFNFVSAIAIGSITAILATNQNFSMLNGI 62
H3
H4
S1
H5
H6
70 80 90 100 110 120 130
Bsubtilis_YetF FASLLWGVLIYIIEFITQKMKSSRKFLEGEPNIVIRKGELQYKVMKKNKIDINQLQSLLR-QAGSFSI 130
Aeribac_WP_063387519 FTVVIWGMLIYLLEFITQKWRRTRGILEGAPSMVIHKGKIIRDQLKKNKLDMNQLQHLLR-AKGAFSI 129
Amphibac_WP_015010493 FAIATWGTLMYVTEFITQRFKGSRSLLEGRPAIIVRKGKIDREAMKKNKLDINQFQHLLR-LKDVFSL 131
Anoxyibac_WP_012575618 FAIALWGLLIYATEMITQKKKELRELLEGKPVIVISKGKILYDALKKTKLDLNQLQHLLR-ARNVFSI 130
Bamylo_WP_013351317 FASLLWGILIYFIELITQKVKSSRKLLEGEPNIVIHKGKLKYDVLKKNKLDINQLQSLLR-QANCFSI 130
Banthracis_NP_844257 YAVFVWVILIYAIEVITQKFRRTRKFFEGYPSIIIRNGHIDREQLKSNHLDINQLQQMLRQQKDIFSI 131
Batrophaeus_WP_003328379 FASLLWGLLIYIIEFITQKLKGTRKFLEGEPNIVIQKGILKYEVLKKNKLDINQLQSLLR-QQGCFSI 130
Bbrevis_WP_012685590 LPMLLIALLEVAFAYLSLKSKKFRDTVDGSADLIIENGQIREQAMRRNRLNMDDLMVHLR-QKDVKNI 130
Bcereus_WP_001015543 YSVFVWVILIYTIEVITQKVRGTRRFFEGYPSIIIRNGKIDREQLSVNHLDINQLQQMLRQQKDIFSI 131
Bfirmus_WP_174752055 FAVTVWGTLIYATETLTQKFKRARKLLEGEPSIVIKKGKIIFEELKKNHLDLNQLQHLLR-SKDVFSI 129
Bhalodurans_WP_010897400 IPIVVLSLIQIVFAYVSLKSKTMRRLVDGKPTVLISQGKIDEREMRKQRYNFDDLLIQLR-QSNVRDI 129
Blicheniformis_WP_003185310 LAFFIFVFVILATAYISLWSQKGRKFFAGDPTVVIQNGKVLEHNMRKMRYTIDYLNQQLR-EKNVFKI 130
Bmegaterium_WP_013057585 YSVLIWGLLIYAVTKLSQTFTGFRGILEGKPSMIIYKGKILYKELKKNNLDLNQLQHLMR-QQGYFSL 128
Bpumilus_WP_041815618 FAVAVWGILIYTVEWFSQRFKGIRAFLEGRPTLVIDQGKIHYNRLKKNMLDLNQLQTLIR-AKGHFAL 129
Bthuringiensis_WP_001015535 YSVFVWVILIYTIEVITQKVRGTRRFFEGYPSIIIRNGKIDREQLSVNHLDINQLQQMLRQQKDIFSI 131
Bweihenstephanensis_WP_002126773 YAVLVWVVLIYTIEVITQKFRGTRSFFEGSPSIIIRNGYIDREQLSSNHLDINQLQQMLRQQKDIFSI 131
Ccandidums_WP_123043699 APIALLVVIQIVFAFLTIRSRKFRLWIDGTPSVLIRGGKLNRSEMRKQRYNLDDLMAQLR-EHEMINV 127
Fphosphorivorans_WP_066393213 YAIVVWGSMVYIVEIISQKFKGTRKFLEGAPTIVIRNGLIDRDMLKKSKLDINQLQNLVR-QKGFFGL 131
Gstearothermophilus_WP_053414763 YSITLWGAMLMTVEYASQKWLSFRLWSEGKPTVLIQNGVIDYEALKKSRLTLNQLQSLLR-KHETFSI 129
Hhalophilus_WP_014641757 IALVSWAVFTVFLGYLDIKSKDVRSFIDGQPLIVVKQGKVLEDELRKARLDLDSLNVLLR-KKNAFSL 129
Lamyloliquefaciens_WP_068444514 FLIALWGLLIYITEIITQKFKGSRYLLEGQPSMVIHKGHLVYDVLKQNRLDIDELQHMLR-SKDVFSI 130
Lsaccgaru_WP_102992088 SPIILLASLQILLSFILIKSERIRNWVDGSPVPLIENGKIVDKNMAKLRYNLDDLLTQLR-EKNIPDV 127
Lsphaericus_WP_024363643 VSLIIWCSLTTLIGYISLKSGKIRMLLDGQPTIVIKHGKIDRKALKRTGVNIDDLTMMIR-QYQVFSI 132
Nthermophilus_WP_077719037 VPVIVLMLLQVSMSFLSLKSSKIRKVIEGTPSYLIKNGEIQEQEMAKHRYNIADLLLQLR-EKNVEKV 129
Oiheyensis_WP_011065627 FVITIYAIIMYISEIITQKYKHTRHMIEGFPSIVIYNGKLIRDTMKKNKLDINQLQHLLR-TKDTFSI 130
Paraliobacillus_WP_112180199 YALAVWSAITIVIGIIDLKSTKFRYAVAGQPRVVIKEGKIMEEELRKVRLDIDALNVLLR-KKNIFAI 129
Pcavernae_WP_119884294 TSLVWWTVLSVGVSLISLKSLKLKKWVDDEPSIVIKRGKIQENVLKKKRLPVEDLLMLLR-LQGVFSF 129
Ppolymyxa_WP_013371121 FALALWCALAYFFEKATTHFVKFGYMAEGRTVLLVDKGQVNQELLEKYDIEFTQLLSMLR-QQNIFSL 130
Pthermoglucosidasius_WP_003250158 YSISLWGALLLLVEYMSQKYLSFRRFSEGEPTVLIRNGIIDDKALKKSRMTLNQLQSLLR-QNETFSL 129
Rstabekisii_WP_066786518 YAIILWTVLSLITNLVVRKYDKLRPFIKGEPSILINKGVLDIKELKKNKMETEQLLSLLR-QQGIFSI 128
Spsychrophila_WP_067207038 IAMVWWAFLTILMSYIAFKSKKARILLDDQPTIVVYEGKIVEASLKKLRLHLNDLGMMLR-EQSIFSV 135
Ssilvestris_WP_014824326 LAFVIYVFIIFMIALISLKSKKGRKFLAGDPTIVIENGKILEKNMNKMRYTLDYLNQQLR-EKDVFNI 130
Sterrae_WP_028976672 FAVALWGLMIYAVEWSTQKFRGTRQLFEGKPTIVIRNGHMDREAMKKEKLDINMLQNLLR-HKNVFSI 129
Tavium_WP_087458819 VSMVIWGGLSYVTEMLAQKSRKIRLLLEGEPTVVIKEGKILEKSLRKEKMNVEELIMLLR-QERIFSL 128
Tcomposti_WP_015255871 YGILIWGLIIYAIGRLTLKSKKMRTLFEGEPSIVIRRGHLQYDVMRRNMLDLDQLLSMLR-QQGYFSL 130
Tgoriensis_WP_038563309 IAIIAIGLFQKAIVKWKISNRKIGKLLTLGPTIVIQDGTLLYENMRKIQYSIDNILFMLR-QKDVFDI 134
Vhalodenitrificans_WP_121616112 IALTGWAGFTLIMGYIDIKSQKARKFTTGIPEIVIKDGKIMEEALRRTRLDNDSLQALLR-QKNVFSM 129
Conservation 30% -100%

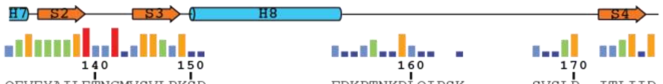


```
Bsubtilis_YetF                    QEVEYAILETNGMVSVLPKSD-------------FDKPTNKDLQIPSK-------SVSLP--ITLIID 176
Aeribac_WP_063387519              REVEYAVLETDGTISVLKKSE-------------FDKPTRNDLNILSS-------PATIP--YTIIVD 175
Amphibac_WP_015010493             RDVEYAILENDGTISVLNNTR-------------SQTPTRKDLKLQDE-------KVYLP--VTLIND 177
Anoxyibac_WP_012575618            REVEYAILETDGTVSVMKKAP-------------YEQPTRQDHNISAS-------EAVLP--VTVIID 176
Bamylo_WP_013351317               QEAEYAILETNGMVSVLPKFP-------------YDTTKNGDMQIKPK-------QVSLP--VTLIID 176
Banthracis_NP_844257              REVEYMILEPNGNISVLKKSK-------------YESPTINDLSLKHK-------PVYLP--ISLISD 177
Batrophaeus_WP_003328379          KEAEYAILETNGMISVLLKSK-------------YDTPKMQDVELAPK-------QVYLP--ITLILD 176
Bbrevis_WP_012685590              ADVEFALLEPTGQMSVFLKEQ---KE----------KVTREDLALMKKLQVGPVSYKGLP--IPLILD 183
Bcereus_WP_001015543              REVEYMILEPNGNISVLKKSK-------------YESPTINDLSLKHK-------PVYLP--ISLISD 177
Bfirmus_WP_174752055              RECEYAILETDGTVSAFKKPL-------------YATPTIQDLNLPIY-------NAELP--VTLILD 175
Bhalodurans_WP_010897400          SDVEFAILEPSGKLSVIEKR----------------PFSGKSSS-------------YLP--LPLILD 166
Blicheniformis_WP_003185310       EEVLYAIVETNGTLTVLKKPQ-------------FRNVTKKDLWVATTPEG------KLP--IELIMD 177
Bmegaterium_WP_013057585          YEAEYVILETNGEVSVAPKHE-------------FGPPTKNDLNIPYS-------QTNLP--IALIMD 174
Bpumilus_WP_041815618             NEIEYAILETDGSVSVLPKMK-------------YATATAEDVNVKGK-------EVKLP--RTFIID 175
Bthuringiensis_WP_001015535       REVEYMILEPNGNISVLKKSK-------------YESPTVNDLSLKHK-------PVYLP--ISLISD 177
Bweihenstephanensis_WP_002126773  REVEYMILEPNGNISVLKKSK-------------YESPTINDLSLKHK-------PVYLP--ISLISD 177
Ccandidums_WP_123043699           EDVELAVLEANGKLSVVPKTDRNSKETGITQRNVSAPSPGKSETPPKIRYE------LLP--LPLILD 187
Fphosphorivorans_WP_066393213     REVEYAILESNGSLSILPKYQ-------------YGPPNRQDLNLQSQ------EQPELP--ITLILD 178
Gstearothermophilus_WP_053414763  REVAFCFLEADGEISVLKKAH-------------YQKTTREDFQLPPH-------PVYVP--VTLIRD 175
Hhalophilus_WP_014641757          SEVSYAIFETDGTLSVMKKTG-------------EQPFTKKDGNVVSN------SPAVYPIPTTVVSD 178
Lamyloliquefaciens_WP_068444514   EEVEYAILEADGEVSVLKKPE-------------YQTPTIADLNLAPK-------PVKIG--RVLISD 176
Lsaccgaru_WP_102992088            SDVEFAVLETTGKLSVFPKEE---KR----------PAFKEDVRRDLK-QF------RMP--IPVIID 173
Lsphaericus_WP_024363643          DEIDFAILEPNGTLSILKKPP-------------FQGTQKIDINISPE------NPPFLP--IEIISD 179
Nthermophilus_WP_077719037        TDVEHAILETSGKLSVILKEG---KQ----------PVTKEEAGVELRGQH------HLP--VPLIID 176
Oiheyensis_WP_011065627           QEVEFAVLEANGTLSVLKKSD-------------YQIPTRKDLKMSTT-------EVDLS--TTLIND 176
Paraliobacillus_WP_112180199      NDVDYAIFETDGSLSVDKKEK-------------VKPLAKTD-LQIQN------KANVFPTPTMIVSD 177
Pcavernae_WP_119884294            QEVAYAVLETNGQLSIMKTEE-------------AKNPTKSDLGVAKK------TPKKIP--AAVFID 176
Ppolymyxa_WP_013371121            REVLYATLETNGSLSVMRKPE-------------YEPPAAQDMGIDAQ-------PDLFS--VTVIDK 176
Pthermoglucosidasius_WP_003250158 REVAFCYLETNGTISVLKKAK-------------YQKTTREDFQLPPH-------PVHVP--VTLIRD 175
Rstabekisii_WP_066786518          REVKYVILEPGGQISVMKR--------------------DSS-DQIT-------TDTLP--RLIIDE 165
Spsychrophila_WP_067207038        KDVHYAILETNGKLSVLKKAG-------------LEAATKKDVNAAIQ------VPKYIP--TEVIAE 182
Ssilvestris_WP_014824326          EEVLFAIIETNGTLTVLKKPQ-------------FRNVNKQDLMIPITPEF------NLP--IELIMD 177
Sterrae_WP_028976672              REVAFAILETDGSISVKQKSQ-------------YATPTMEDLQKQQK-------PVYLP--ITIIED 175
Tavium_WP_087458819               TEVDYAILEPDGQLSILKKTK-------------HLPASKHDVETGRA------NTAPFG--VQIVSD 175
Tcomposti_WP_015255871            REIEYAVLETNGMISVLPKRP-------------YGPPTRADLGLHGV-------GGRVP--ITLILD 176
Tgoriensis_WP_038563309           RDVETAIVEPNGALSVLKKQS-------------KMPATREDLQIQQQ-------PSAIA--FPVISD 180
Vhalodenitrificans_WP_121616112   KEVDYAIFETSGKLSVMKKEN-------------KQPATKSDIHQLTTY-----SPKIFP--TEIISD 177
```

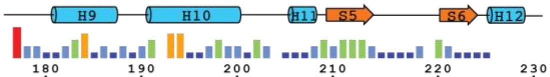


```
Bsubtilis_YetF                    GEIVRDNLKEAGVDEQWLKQELKKKNIDKTEDVLFAEWHKNKP-LYTVTYEQSRST          231
Aeribac_WP_063387519              GEVVSENLKEAGFDEKWLQQQLLAYSVEDAKDVFYAEWDQNNG-LFVQKY                224
Amphibac_WP_015010493             GEIIEDNLAEINKDKDWLYDQIKKQKISSIEEIFYAEYLEDEP-LFIQKM                226
Anoxyibac_WP_012575618            GEVIWDNLRENGWDEQWLKKHIRHAGFENYSDILYAEWQDGKG-MHVQPY                225
Bamylo_WP_013351317               GEVLYENLKEAGVKEEQLIQDLKKQNIKRTEDVFYAEWKHDKP-LFVLPYEEKK            229
Banthracis_NP_844257              GKVVKDNLREAGFDEGWLYKQIKKKGITKFEDVLYAEWKTDDG-FFCQEMDR              228
Batrophaeus_WP_003328379          GEVVYDNLQEAGVDEEWLKQELASQGVNECKDVLFAEWHAGQP-LFFIKYDSGSDK          231
Bbrevis_WP_012685590              GKVRTEALSKIGQNELWLKREIRKYGIKDIREVSFCSIDE-RGIMYLDKKDKP             235
Bcereus_WP_001015543              GKVVKDNLREAGFDEGWLYKQIKQKGITKFEDVLYAEWKTDDG-FFCQEMQR              228
Bfirmus_WP_174752055              GEVVWDNLKSINWDETILKNEIKKYGASGVKDVLYAEWKKGEA-LHVQTY                224
Bhalodurans_WP_010897400          GKVQEKHLEKINKTSLWLRQQLRKLGYRDIKKISYCAIKE-DGTFFIDIKDEK             218
Blicheniformis_WP_003185310       GKMKNENLKDNQLTQAWLQSEMKKRGLSTDEVFYAVLSPD--GNIYIDTYEDHIHSSFDKE     236
Bmegaterium_WP_013057585          GKVVPGNLKEANVDEKWLKKQLAIKKIKKYSEVFYAEWQQERG-LEITKF                223
Bpumilus_WP_041815618             GEILKDDLQEAGFDETWLSEQLKRQHISSYRDVLFCEWIENEG-IYAMKYEKSKKHS         231
Bthuringiensis_WP_001015535       GKVVKDNLREAGFDEGWLYKQIKQKGITKFEDVLYAEWKTDDG-FFCQEMQR              228
Bweihenstephanensis_WP_002126773  GKVVKDNLREAGFDEGWLYKQIKQKGITKFEDVLYAEWKTDDG-FFCQEMPR              228
Ccandidums_WP_123043699           GQVQDDSLEKIGKNRFWLKTRLREQGIEQFRQVFFCSIDH-KGRFYVDRKK               237
Fphosphorivorans_WP_066393213     GELNHDNLSTAGLSRNKLLEQIHKQGYSDIKEVVYAEFSEGK--LLIMPCTKPKK           231
Gstearothermophilus_WP_053414763  GQLLADELAELGKTEQWLAAKLQKQGIASPKDVLIAEWLEGDG-LFVQTYQPAERQRPTRRQTAPE 240
Hhalophilus_WP_014641757          GKINDANMQSLNLDQKWLHHQLKSAGVPSISDVFYAEVQKDGS-LYIDKRNDTIH           232
Lamyloliquefaciens_WP_068444514   GEIVWDNLKEASLNKTWLEEELHKQNVQSIEDVFYAEWQENQQKLFVALYHTKKKRQN        234
Lsaccgaru_WP_102992088            GKVQEEGLKKLGQNRFWLKAEIQKRGLKDFKEVFYAAVNY-EGRLYIDRKDRPTR           227
Lsphaericus_WP_024363643          GKLLKRNLLEVGKNRQWLKDELKKMSIENIEEVFYAEIQSNGQ-LFIQKY                228
Nthermophilus_WP_077719037        GKVQDKRLEEIGQTRFWLKNEVQKQGYKDFKDIFFALYDVSNGELHIDRKQS              228
Oiheyensis_WP_011065627           GEVIQDNLKEKNLSIEWLMEQIKKEGYERIEDVFYAEYIKDKP-LFLLPYINRNHQKWDVD     236
Paraliobacillus_WP_112180199      GKIDNNNLEKLHLNKDWVMKQLESAGVGSIDEVFYAAVQKDGT-LYIDNTNDTVH           231
Pcavernae_WP_119884294            GNWMTDACHEIGVAQKTIEKQLKDSGYKNLDDVFLVQWQEDGT-LYVDKKTDVKQS          231
Ppolymyxa_WP_013371121            GRLLNESMRGKTIDVELIKAKTREQGYDSIDEIAYAELSEDGT-LHIVPMKEK             228
Pthermoglucosidasius_WP_003250158 GELLRDELQELGKDEQWLNEQLRAYGVTSHQDVFIAEWLEGDG-LFVQTYS               225
Rstabekisii_WP_066786518          GQIVNRTLNQIRKNEQWVINLLKKEGYEDIEKVYYAEWSEGKG-LYVQCNE               215
Spsychrophila_WP_067207038        GKISKGNLAELNLTEEWLFEQLKKQGIGNVKTVFFAEVQTDGS-LHVDMKAEGEK           236
Ssilvestris_WP_014824326          GEVMKQNLDQNNLSEQWLHSELKQRNLVQSNVLYAVLSGN--GNMYIDTYENHIHSPIDKE     236
Sterrae_WP_028976672              GEVDWDNLKEAGLNEEWLLKNLRRHHIRSYKDVFYCEWKKDEG-VFIEKMNANERR          230
Tavium_WP_087458819               GQLLTHNLKTIKRDEKWLEQQLKQQGIPTYKEVFYAEAQEGGG-LYVDRKKDQPY           229
Tcomposti_WP_015255871            GRLQSDNLGKASLSEPWLKQQLADRGITSYEGVLYADWDPEEG-LFVMPHQ               226
Tgoriensis_WP_038563309           GYIHHDTLTHFKLDEEWLRKQLAEQNIIGMNQIFFASISHPQS-LHISLKERKLNIPPIKH     240
Vhalodenitrificans_WP_121616112   GELNEANLKKLNLDTNWLNGQLKQAGITSLSEVFYAEVQPDGT-LYFDNKRDGLYH          232
```

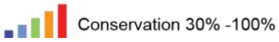

Supplementary Figure S5

Sequence alignment of *B. subtilis* YetF with the best YetF match in other *Bacilli*. Sequence conservation is shown as a bar graph, with red bars indicating identity among YetF homologs. Secondary-structure assignments of YetF from the crystal structure (PDB ID: 3C6F) are shown as blue cylinders (helices) and orange arrows (β strands). Predicted secondary-structure elements by AlphaFold for the N-terminal TM region are shown as gray cylinders (α helices).

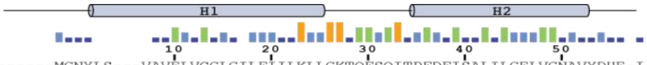


```
Bsubtilis_YetF                              ------MGNYLS---VAVELVCGLGILFIILKLLGKTQFSQITPFDFISALILGELVGNAVYDHE-I 57
Rcellulolyticum_WP_015924636                --MN---EGL----VVFVRSLLSFFSLLIFTRILGKEQISQLTFFDYILGITIGSIAASLSTDLS-S 57
Sglycolicus_WP_013625014                    ------MIIA------TIRTIILYLVIVIIIRIMGKKQIGQLQPFELVIILMISELAAIPSQNIG-I 54
Sthermophilum_WP_011197382                  --MG---EVS----VVLVRSAITFFNLLILCRLLGKTQVSQLTFFEYVSGITLGSLAAELSTNLS-L 57
Tacetatoxydans_WP_013778043                 ------MSILKDLLLHAYKSLILFMVSLTLVRLMGKRTVAQLSPFDLVFMIIMGSAIAIPLEDHD-I 60
Titalicus_WP_012994581                      ------MLIL------FFRTLILYALIVIFMRISGKQQIGQLQPYELVVAIMIADLVAIPMQNKG-I 54
Tphaeum_WP_015049695                        --MTDALLYL----QMALRAVIAFLAILLIARILEKEHVGELSLYEYLTGIAIGVIAGRIATTMQ-N 60
Tstercorarium_WP__015358013                 -----MFFDI------VWKTALMYLVVIVSMRLMGKRMIGELQPFEFAIAIMISELAVFPLTEGN-T 55
Tthermosaccharolyticum_WP_015312322         ------MLII------FFRTLILYFLVVVVMRIMGKQQIGQLQPYELVVAIMIADLVAVPMQNKG-I 54
Adegensii_WP__015739924                     --MN---PFL----EILLRAVGAFLGVLFITRLVGKSQVGQLTISDYVNGIVIGSIAACLATDIK-E 57
Adegensii_WP_015739458                      ------MSVLG---QVVWRTVLIYFVVLVMMRLMGKREIAQLSPFDFAVAIMIAEVAVLPMESLE-I 57
Ametalliredigens_WP_012065355               ------MIVI------FIRAFVLYMIVVIVMRMMGKGQIAEMQPFELVITIMIADLAATPMENTS-V 54
Athermocellus_WP_003516115                  ------MLII------VVRTLILYILVVIIMRIMGKRQIGQLQPFELVIAIMISELAAVPMQDKR-I 54
Cacetobutylicum_WP_010890760                --MED--PLI----NTVFRSVIVYLIAMFLTRLMGRKLISQMTFFDFVMGVSMGSIITDSIVGQK-F 58
Cacetobutylicum_WP_010964829                --MN---EGL----VVIVRSIIGFFTMLIFAKILGKQQISQLTFFDYILGITIGSIAATLTTDLS-S 57
Caproiciproducens_WP_128742257              ------MTIS------FIRTVLLYAVVILAVRLMGKRQISELQTSELVVTLLISDIASIPMQDSG-Q 54
Carthromitus_WP_005805303                   ------MLIV------LSRTIIIFVVMFFTMKMMGKRYIAQLEPYEFVVSIMIAELATLPLEDVS-I 54
Cbotulinum_WP_011948896                     --MN---EAL----VVLVRAIIGFFTLLIFARMLGRQQISQLTFFDYVLGITIGSTASTLSTDLE-S 57
Cdesulforudis_WP_012303154                  --MN---PFL----EIVIRAVGAFIAVILITRLVGKSQIGQLTVTDFVNGIVIGSIAAALAIDIR-S 57
Cdifficile_WP_009889750                     ------MTVV------LIRSIILYITVLIALRVMGKGEIAEMNCFDLVITLLIAEVASVPMENNN-I 54
Clentocellum_WP_013658886                   ------MEVI----QAIITALGSAAALFTLSKLMGNREMSQLSMFDYINSITIGSIAAEMATCEF-T 56
Cperfringens_WP_003472738                   ------MLIT------FIRTAILYFIVIISIRLMGKRQIGELQPYELVITLMLSDLASLPMQDTR-L 54
Csubterraneus_WP_009611278                  ------MLIL------FFRTLILYALVVVFMRISGKQQIGQLQPYELVVAIMIADLVAIPMQNKG-I 54
Ctetani_WP_035111051                        --MN---EGL----VVVIRSIIGFFSLLIFARVLGKQQVSQLTFFEYVLGITIGSTASTLSTDLS-T 57
Dacetoxidans_WP_015757363                   ------MLLI------LIRTLILYLGVVIVMRVMGKQEIGQLQPYELVIALMIADLAAIPMSNTG-V 54
Dformicoaceticum_WP_089609402               ------MET-------VLRTVGLLAFGFICYRLMGYRSMGDMEPTDFVIMLVIAETLGTPLADES-L 53
Dgibsoniae_WP_006522671                     MLIN---PYI----EIPLRALFAYLFLLLFTRINGREQISQLTYFEYVVGITIGSIAGTLTTTPE-D 59
Dhafniense_WP_011459753                     --MS---EGL----VVIVRSLIGFFSLLIFARIIGKSQISQLTFFDYVLGITIGSMAASLATDLS-S 57
Dkuznetsovii _AEG16849                      --MN---PYL----QILIRGIGAFLGVLVITRVVGKTQVGQLTVADFVNAIVIGSIAAAMVTDLK-E 57
Dorientis_WP_014187430                      ------MLIV------VVRTLILYALVIIALRLMGKREIGQLQPFELVVILMISELAAVPSENVG-V 54
Druminis_WP_041275606                       ------MALT-----YVWRTVIIYFSVLVIVRLMGKREIGQLSSFDFVVAIILAELAAIPMESEK-I 55
Ibutyriciproducens_WP_058117823             ------MIIA------FLRTIILYLLIIAGIRLMGKRQVGELEPSELVLDLIIADLAAVPMQDFG-I 54
Lphytofermentans_WP_012198749               ------MTII----RVILSSFASIVALFFLCKFIGYRQMSQMSLFDYINGITIGSIAAEMAT-DL-E 55
Lumeaense_WP_111524166                      ------MNAFLL---VAIKLLIGFFALVIIINISGKGNLAPSSASDQIVNYVLGGIIGGVIYNSS-V 57
Maustraliensis_WP_013780881                 --MK---MIA----EIIIQTIMAFFAILIFTRLLGKQQITELTYYDYINGITFGSIAGVMATDIS-Q 57
Mtermoacetica_WP_011391764                  --MK---FV-----EVFLQTLLAFFAILIYTRILGKQQIGQLTFFEYINGITFGSIAAVLATDTAPN 57
Psordellii_WP_055331643                     ----MINEIY----IVSIKSIISVSILFILTKIMGKKQISQLTLFDYVVGISIGSIAAELAINES-I 58
Pthermosuccinogenes_WP_103079909            ------MLVV------FVRTLILYAVVIVVMRLMGKRQIGELQPFELVVAILISDLAAVPMQNTG-I 54
```

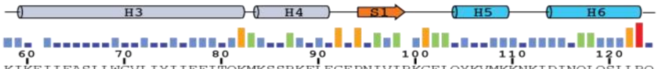


```
Bsubtilis_YetF                              KIKEIIFASLLWGVLIYIIEFITQKMKSSRKFLEGEPNIVIRKGELQYKVMKKNKIDINQLQSLLRQ 124
Rcellulolyticum_WP_015924636                RSWPHWIGLITWAALGLLLEYITLKWRYVAKYLENEPTIVIMNGKIMEKTLKKMRFRVSDITELLRN 124
Sglycolicus_WP_013625014                    PLSAGLLPVIVLLLLGMTFSYITLKSEKARALICGTPTILIEKGKIIEKELVDLKYNLSDLLEQLRV 121
Sthermophilum_WP_011197382                  RPWPVFVGLFAWGGFTLLTQLVALKSRWMAKMLDGEPVILVYRGQILEENLRRLRMRESELAELLRS 124
Tacetatoxydans_WP_013778043                 RLTHGIFPVAITSLLNYLLAIIITKNRKVENILQGTSRILIKDGEVVVSNLKKERITITDLLVLLRE 127
Titalicus_WP_012994581                      PLLSGIIPILTLLVSQLFLSYLSMKSLRARAIICGTPTILIEKGKILTSQLQKERYNINDLLEELRV 121
Tphaeum_WP_015049695                        -PLPLLLALFVFAALTYLVRYFTLKSRAVRKLLAGEPAVVIQNGKIMERTMRRMHYSTDDLLMQLRT 126
Tstercorarium_WP__015358013                 NILHGLLAIGVLVLCQFLLSLLSIKSLTVRTIICGRPRIVIKNGKILEKNLKKELYTINDLLEQLRM 122
Tthermosaccharolyticum_WP_015312322         PLLTGIIPIFTLLISQLFLSYISMKSLRGREMICGKPTVLIDKGKILTKELQKERYNINDLLEELRV 121
Adegensii_WP__015739924                     NPWYYVFGLAIFASLTILVQYVSLIYRPARKFLLDEPTVVVHNGRILERNMARMRYNVDDLMSQLRE 124
Adegensii_WP_015739458                      PLFRGLLPLLILTVLEIGFSYLSLHSRWLRRLVYGEPQLVIWEGKVLHREMRRARYNLDDLLAQLRE 124
Ametalliredigens_WP_012065355               PLINGVIPILTLLSGQVIISYLSLKSTKFRGLICGKPSILIHKGRIVQSELRRLRINVNDLLEQLRS 121
Athermocellus_WP_003516115                  PLINGIIPILTLLFAQITFSFIGLKSTRARAIICGRPSILIQNGKINEAELRKEMYSVNDLLEQLRI 121
Cacetobutylicum_WP_010890760                TSISSVTALTVLSILVIITGYIHIKSLKLREIINSKPIILVDNGNIVEENMKESRITINELLMKLRE 125
Cacetobutylicum_WP_010964829                RAWPHWVGLLTWALLGYLMEYISTKWRYAGKYLEGEPTIVIMKGKIMEDAMKKMNFRATDIMELLRD 124
Caproiciproducens_WP_128742257              PLASGLIPIAALVMFEIVLSVLMLKSTKFRKLICGRPIIVINNGVVQQNEMKRLRMTTEDLSEQLRQ 121
Carthromitus_WP_005805303                   PLLYGIICIFTILFIEFIISQIQLKNIKLRKLFGGKSVILVKDGKFIKENLKNEKLTINDVLEDLRS 121
Cbotulinum_WP_011948896                     TAWSHWIGLLTWCAIGFLLQWITLKWRYAAKYIEGEPTIVIMDGKIMEDTLRKMKYTVADVLEQLRG 124
Cdesulforudis_WP_012303154                  PASFYFLALAVFSGLTLFLEWSTMKSRPVRKILEDEPTVVVHNGKILETKMRNMRYHLDDLMMQLRS 124
Cdifficile_WP_009889750                     PIINGVAAISGLVIMQTLISFLSLKSRKLSSFLSGKPSVLIDKGKIVYKELKKERISIDELLEQLRI 121
Clentocellum_WP_013658886                   DLLKPFTAMIVFALFNILLSLLTNKSIKIRRLATGKPSILYDNGQLYYKTFAKAKMDLGEFLVQCRV 123
Cperfringens_WP_003472738                   PLLLGIVPIITLLFVKILISEIQQHSRLFEKILDGTPSIIISDGEINLEMMKKQRLTMNDILEELRS 121
Csubterraneus_WP_009611278                  PLVAGIIPVLTLLISQLVLSYLSMKSLRARVIICGTPTILIEKGKILTSQLQKERYNINDLLEELRV 121
Ctetani_WP_035111051                        RAWPHWVALISWAILGYIMQLITLKQRTMAKYIDGEPTIVIVNGKIMEEALKTMKYRAADILQLLRN 124
Dacetoxidans_WP_015757363                   PLANGLVPIFGLLIAQVFISYAALKNIKFRTIVCGAPSILVKNGQLVESELRRIRYNIHDLLEQLRV 121
Dformicoaceticum_WP_089609402               SLTNTIIAVSTLTILQILFSWTGLKSTFLLQLMDGKPIPVIQNGKILEKNMRKARVNRSDLMEELRV 120
Dgibsoniae_WP_006522671                     PFGPGLLGMAVWTILPILTGLLVLKSVPARKIIEGEPIVVIQNGKIDEEALARQRTNLDDLMLMLRQ 126
Dhafniense_WP_011459753                     RAWPHWVALITWAALGYVMEKITIKWRYAAKFLEGEPVIVVMSGKIMDDALRKNNYRISELMGLLRN 124
Dkuznetsovii _AEG16849                      NGWYYAFGLLLFGLLTVISEYASLKYRPLRKLIEGEPTVVIHNGKILENNMKKLIYNVDDLMMQLRE 124
Dorientis_WP_014187430                      PMLSGVIPILVLLSASLTLAWISLKSEKARNIICGKPSILIDRGKISQDELRKNCYNITDLLEELRL 121
Druminis_WP_041275606                       PIWHGIVPIATLGLLEVTFSYLTLLNRPLRKIVYGSPQVIIENGKLLKHEMRSSRYNLDDLLSQLRE 122
Ibutyriciproducens_WP_058117823             PLLSGIIPILTLLSVTMVLSVLTMKSVKFRAIVCGRPSIIVENGRLNQQEMRRNRFTVDELNEELRV 121
Lphytofermentans_WP_012198749               NPLHALIAMAIYSVVAVALSIGTDKWLGFSRFVNGYPLVLLDNDKLLYENFKKAKIDIEEFQIQCRN 122
Lumeaense_WP_111524166                      RILDFIAILCIWCILVLGLKWIKTHNVKAKQLIDGKALTIIDGGKIIVENCKKAGLSAHDVSFKLRT 124
Maustraliensis_WP_013780881                 RTRQHLMGLALFALLTFLMSYISMKSRPARKLLEGEPTIVVLNGQILEDNLRKMHYNIDDLISELRQ 124
Mtermoacetica_WP_011391764                  QTWMHFLGLTLFAFFTWLAGYAVLVSRPARKLISGEPTVVVHNGKILEENMKKMRYNFDELAMQLRQ 124
Psordellii_WP_055331643                     SYIQGITGMTIYALFPILLSFISLKSHLARKILDGVPTILIQNGEINEKGLKKTKMNINDLIEECRL 125
Pthermosuccinogenes_WP_103079909            PLVNGIIPIFTLLIAQLAMSLISLKSVKARGIICGKPSILIEKGKINESVLRREMYTINDLLEQLRS 121
```

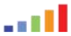 Conservation 30% -100%

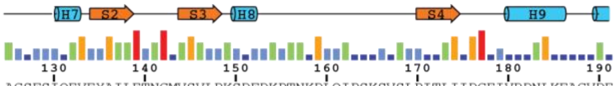


```
Bsubtilis_YetF AGSFSIQEVEYAILETNGMVSVLPKSDFDKPTNKDLQIPSKSVSLPITLIIDGEIVRDNLKEAGVDE 191
Rcellulolyticum_WP_015924636 KDIFDLTEVDFAIIEPNGQLSVLKKPEYQNLTPKDMYIQKKASGISSELIYDGILIEENLRQLDKDK 191
Sglycolicus_WP_013625014 NNIPNIADVEYAILETNGQLSVIPKTDKRPLTPADMNIHPAYEGLPSILIMDGIVQKRNLERSNANL 188
Sthermophilum_WP_011197382 QSVFHFDEVELAVLEPRGTLSVLRTADTQPVTPADLGIPASSRGLGIELVVDGEVMDQNLRRLGVNR 191
Tacetatoxydans_WP_013778043 KDVTNINEVEEAAIEPNGKLSVIKKKYMQTVTPRDLGLWSNQGIFPTLVIDGGEVIQDNLDRVGVSI 194
Titalicus_WP_012994581 KGYPNIADVEYAILETNGSLSVIPKSDKRPVTPQDLNLTPQYEGLPLPIIIDGRIMHQNMQKAGIDM 188
Tphaeum_WP_015049695 KGVFNISDVEFAVVEPNGRLSVLLKSQKMPVTREDMQIPSQYRGISSELIVDGEVIYQNLRQNNLDE 193
Tstercorarium_WP__015358013 LNIQNISDVEYGILETNGQLSVVLKSQKRPVTPEDLGIETKYEGLSLDLIIDGIVITHNLKLAKLDM 189
Tthermosaccharolyticum_WP_015312322 MGYPNIADVEYAILETNGCLSVIPKVDKRPVTPNDLNLTPQYEGLPLPIIIDGKIIHKNMKMANVDM 188
Adegensii_WP__015739924 KGYFNLADVEFAIVEPNGSLSVLPKSQKRPVTPEDLGLPTKYEGVPSELIVDGQIIYQNLVQNNLTE 191
Adegensii_WP_015739458 KGYHDPSEVACAVLESSGRLSVIPRSEYRPLTRGDLGLPPVPVGPVRVLVADGEILEENLKVVGVDR 191
Ametalliredigens_WP_012065355 KEYLNLSDVEYAILETNGQLSIMPKPDKRGVITSDLAVAVQQEELPVTLIIDGKVNQNNLKRAGYDM 188
Athermocellus_WP_003516115 KNIYNIADVEFALLETNGQLSVIPKSQKRPVSPEDLNIPTKYEGLSLDIIVDGEISHENLKLANLDV 188
Cacetobutylicum_WP_010890760 KNIFNMADVEFAIIEIDGQLSVLPKASQKPLTPSNMNINVTSTGLTQDIIIDGNILDENLYKAGLDI 192
Cacetobutylicum_WP_010964829 KDVFDLNEVDFAIIEPNGKLSVLKKPELQPVTCNDMNITKNEMGISTELIYDGILIEENLRQLNKTK 191
Caproiciproducens_WP_128742257 KNVFSIQDVAYAIVETNGKMSVIKKPDKEQPTAGMLAVPLPDTGIETVVISDGVISDFSLQLCGKSA 188
Carthromitus_WP_005805303 GGNYDLSKIGFAILERSGKIAVIPK-NNEN----------EKVYLPTSIIIDGEIVKEGLKYINRNN 177
Cbotulinum_WP_011948896 KDIFDLSKVDFAILESDGQLSVLKKPGEEPLTAKDLNIFKSKTGISRELIYDGEIVEDNLREINRDK 191
Cdesulforudis_WP_012303154 KGVFNIADVEFAVLEPNGEMSVQLKSQKRPVTPADLQLPTRYEGMPSELIVDGTVIEQNLIQNNLDE 191
Cdifficile_WP_009889750 QGYFNLKDVQYAILETDGNLSVVPASSYNSTPPR------AFNHLPIPLILDGRIINKNLDIAQKDT 182
Clentocellum_WP_013658886 NDYFDLTDIQTALLEPNGKISFLPVSNKRPITGEDLNISPSQNYLVANVIIDGKIMLQNLKHIGNNE 190
Cperfringens_WP_003472738 AGYLDISDIQYAIIETNGTISIIPKSACDNVKRKDLKIKESESKIPIVLFEDGRLNKKALQGMNKDE 188
Csubterraneus_WP_009611278 KGYPNIADVEYAILETNGSLSVIPKSDKRPVTPKDLNITPEYEGLPLPIIIDGRIMHQNMQKAGIDL 188
Ctetani_WP_035111051 KDIFDLKQVKFAIIEPNGQISVLKKEEYLPVTPQDMNISPSPSDISIEVIYDGLIIEEHLKWFKKDK 191
Dacetoxidans_WP_015757363 KNIANIADVEFAILETGGKLSVIPKSQKRPLIPEDLQISTKYEGIPTTLIIDGQIMSNNLRMIKLDE 188
Dformicoaceticum_WP_089609402 QGLSNYLDVEFAYLEPSGRFSIIRKKEVEPITPRYLG-----KKTSKTIMENGEIFADQLTQSGVTA 182
Dgibsoniae_WP_006522671 KDIFNIGDVENAIFERNGELTVQRKSQLNPVTPADLNISTLYQGLPTTLVQDGVVIENRLKEISLSK 193
Dhafniense_WP_011459753 KGIFDVSQVDFAILEPNGQLSVLQKPENLPLTPKDMNIKASSSGISSELIYDGMIVEQNLRQFKKDK 191
Dkuznetsovii _AEG16849 KNVFNIADVEFAVAEPNGGLSVLLKSHKQPLTPSDMQIPTKYQGIPSELIVDGVVIQQNLKQNNLTE 191
Dorientis_WP_014187430 NNMPNIADVEFAILENNGQLSVIPKTNKRPTIPDDFKISPQYEGLPLTVIMDGKLNKKNLEHYNKDI 188
Druminis_WP_041275606 KGYPNMEDVEYAILETSGKLSVIPRSQKRPVTPEDLGIPTPYEGLPTVLVMDGDVLEENLQKVNLTE 189
Ibutyriciproducens_WP_058117823 QGITDLSTVKYAILETNGRVSVIPYANQRPVTAEQMNLFPDDVGLPLVIINDGRLLEHNLKLRGFNE 188
Lphytofermentans_WP_012198749 CGYFDITTIQTALLEPNGTVSILPKATHRPATPKDLSIKAPQDYMVFNLVLDGIMIVSNLKILDLDE 189
Lumeaense_WP_111524166 NRIYSTKDVKRAVLEQNGQLILIQSG--EENPK-------------FPVITDGQVQSDILEVIGKDE 176
Maustraliensis_WP_013780881 KDVFNLKDVKYAILEPSGNLSILQNADIKPLTPKDMNISPQQQSLPSELIVDGQIIYQNLAQNNLNG 191
Mtermoacetica_WP_011391764 KNVFDIADVEYAIMEPDGDLSVLLKSQKRPLTPSDLKLSTKYEGVPTELIEDGEILFQNLRQNHLDE 191
Psordellii_WP_055331643 KDVFDIKDIKFAILETSGNLSIQLKSKHKPLTPEDINLKLKDKYLCVNLIIDGKILDNHLEIIGKDI 192
Pthermosuccinogenes_WP_103079909 KDIYNIADVEYAILETNGQLSVLPKAQKRPLTLQDMNIPAQYEGLPLVLINDGYVSKKDLDKAGVDE 188
```

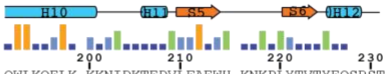


```
Bsubtilis_YetF QWLKQELK-KKNIDKTEDVLFAEWH-KNKPLYTVTYEQSRST 231
Rcellulolyticum_WP_015924636 NWLLNELK-KQGITDISDVFIATLN-PSGSLYIDLYNDHMTK--ITDIGDY-KG----PY 242
Sglycolicus_WP_013625014 TWLKNELN-KANL-KVNDVLIASLD-NQNHLYFQAKKS-----NS 225
Sthermophilum_WP_011197382 TWLMAKLK-EQGIRSPAEAFLAVVD-ADGKLYVDRYTDRVPK--SHDLSDY-PG----PN 242
Tacetatoxydans_WP_013778043 DLMLRQLN-QKGIGRLKEVKSAWID-EDGNLSIERVTETTSK 234
Titalicus_WP_012994581 EWLNEQLK-MWKIQNVKEVLFASLD-TNKVLTVYRKED 224
Tphaeum_WP_015049695 AWLISELA-KQGIKSPREVMLAGLD-TQGNLYVDKRQDDQRH--VVQVRDD-PG--E 243
Tstercorarium_WP__015358013 DWLVGKLK-ENGWDNPRDIFYAYID-TAGNFQFQPKLKASKSRVMNTP 235
Tthermosaccharolyticum_WP_015312322 KWLNDQLK-MWNVNNIENIILASLD-PNKVLTVYKKE 223
Adegensii_WP__015739924 EWLLKELE-KQGVKSLKEVLYASLD-SEGKLYIDKRQDKLEH--LTDVTDKLPG--QSGQ 245
Adegensii_WP_015739458 EWLLRELE-KQGFREPEKVFLATLS-PQGKLFVSPKEEEEGELPN 234
Ametalliredigens_WP_012065355 SWLLKQTE-NEGIKGLHNVFFAFIS-SEGNFHAQEKAK 224
Athermocellus_WP_003516115 NWLNDTLS-KFGINNPKDVLLASLD-SEGKLYYQLKERRNN 227
Cacetobutylicum_WP_010890760 DWLLSQLN-SQNIKNESEVFYAGID-NTKKLYVSKKARKNTKY 233
Cacetobutylicum_WP_010964829 DWLFKVLE-SRGIKNVSEVFLATLN-PGGSLYIDLYKDHIKK--VLDIGDY-KG----PY 242
Caproiciproducens_WP_128742257 DWLQGVLN-GQHLQAG-EIFLMTAN-TKGDFFIVKREAPK 225
Carthromitus_WP_005805303 DWLMKQLK-ENKIKSPKDVLYAYTD-SNGTFKYQLETK 213
Cbotulinum_WP_011948896 EWLKAELK-KRNIKDSSDVFLATIN-ENNQIYIDTYKDHLKR--IIDIGDY-KG----PY 242
Cdesulforudis_WP_012303154 EWLYRELE-KQGVRSSEEVMYASLD-SEGKLYVDLKEDAIEH--YTDITDKVPD--KIPQ 245
Cdifficile_WP_009889750 NWLMGILK-SNHIETFKDVLICVLD-ENDKIFIQNKKGD 219
Clentocellum_WP_013658886 KWLTKQLR-IHDVSDISEVFLATCD-RNNKLCVYKKLTEKMTADLLE 235
Cperfringens_WP_003472738 KWLDEKLK-SLNYPPRDKLFLVMMD-SNGKLFIQRKNQKDKEDIIL 232
Csubterraneus_WP_009611278 AWLDEQLK-IWNIKSVKEVLFASLD-SNKVLTVYKKEG 224
Ctetani_WP_035111051 KWLKKELK-KRNINDPSEVFLATMD-NTGNFYADKYNDLMKKENVTKDGTY-KG----PL 244
Dacetoxidans_WP_015757363 NWLERELA-KFGIDSHQKVLFASLD-TNGKLFWQLKSD 224
Dformicoaceticum_WP_089609402 AELAEILE-SFHIDDLNQLESIVIT-PDGHIALTKKQQ 218
Dgibsoniae_WP_006522671 DWLLKKLQTEHGVNEISQVSIAQLD-TSGNLYVDLKNANPEN--KPH 237
Dhafniense_WP_011459753 KWLQKELQ-KQGIKDVSEVFFASLN-PAGSLYIDLYKDRLQN--PVDIGDY-KG----PY 242
Dkuznetsovii _AEG16849 DWLYRELE-KQGIKSVKDVMYASLD-AEGKLYVDRKEDTMQH--VTDITDKLPG--KMPQ 245
Dorientis_WP_014187430 QWLKKELK-KQKIHQIEDVFIASLD-SSGNLFAQEKKQPKKQKNNKKKKNPDVR 240
Druminis_WP_041275606 AWLKEKLA-ERGFH-PKKVLLATLN-TNGQLLIDCQNDDRQK 228
Ibutyriciproducens_WP_058117823 GWLNKRLE-EHGVRAVRDVFLLSVD-EQNRVYFVPKEVGVK 227
Lphytofermentans_WP_012198749 DWLIKQLK-SQGYNDYHELLLVTCN-HKHEITAYKRYKKNHAS 230
Lumeaense_WP_111524166 DWLLEELK-KQGIEKYSDVFLGEYV-NNSLILTIDK 210
Maustraliensis_WP_013780881 KWLMDMLA-AHDIHSIKEVAYASIDPLTKEFYVDTYKDSVPK--NIDISDVYKG--KLE 245
Mtermoacetica_WP_011391764 KWLIQQLQ-AQGIQDISQVDYAVLR-SNGTLYVNTKEDDIIN--PVDITDAPESPVKTEKEEQDRP 253
Psordellii_WP_055331643 KWLNSELD-KKGIKNTSDILLAYMD-SSKKINIYLKNKDIPITPTL 236
Pthermosuccinogenes_WP_103079909 NWLKSELA-KHGISDARDVLFASLD-SSGNLFLQRKER 224
```

Conservation 30% -100%

Supplementary Figure S6

Sequence alignment of *B. subtilis* YetF with the best YetF match in *Clostridia* species. Sequence conservation is shown as a bar graph, with red bars indicating identity among YetF homologs. Secondary-structure assignments of YetF from the crystal structure (PDB ID: 3C6F) are shown as blue cylinders (helices) and orange arrows (β strands). Predicted secondary-structure elements by AlphaFold for the N-terminal TM region are shown as gray cylinders (α helices).

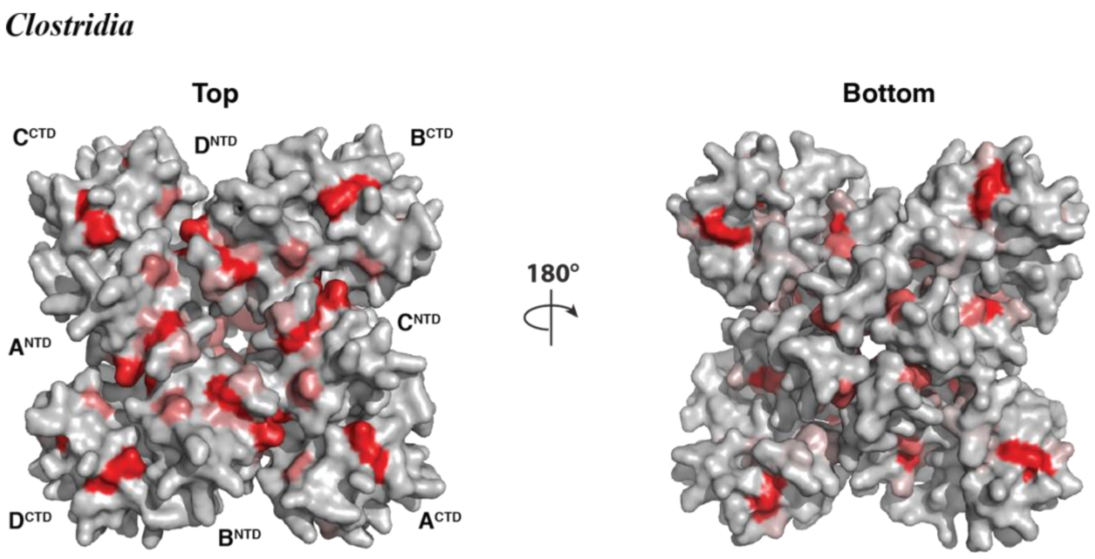

Supplementary Figure S7

Surface representation of a tetrameric YetF, showing conservation among best matched YetF homologs identified in *Clostridia* species.
